# Validation of TRPA1 and TRPV1 Antibodies for Expression Detection in Mammalian Cells and Tissues

**DOI:** 10.1111/jnc.70444

**Published:** 2026-04-22

**Authors:** M. de las Casas, P. Hernández‐Ortego, R. Torres‐Montero, E. De la Peña, A. Gomis, F. Viana, J. Fernández‐Trillo

**Affiliations:** ^1^ Instituto de Neurociencias Universidad Miguel Hernández‐CSIC San Juan de Alicante Spain

**Keywords:** immunocytochemistry, immunohistochemistry, TRP channels, western blot

## Abstract

Antibodies are key reagents in cell biology and biochemistry research. The validation of their performance, in terms of sensitivity and specificity, is essential for their correct application. TRPV1 and TRPA1 are non‐selective cation channels expressed in primary sensory neurons, where they mediate the detection of diverse physical and chemical stimuli. They play key roles in nociception and inflammatory processes, making them important targets for mechanistic and therapeutic pain studies, highlighting the need for reliable evaluation of their expression. The detection and quantification of TRPV1 and TRPA1 protein expression is commonly carried out using antibody‐based techniques, such as immunohistochemistry and western blotting. However, as with other TRP channels and membrane proteins, antibody performance is frequently suboptimal, leading to potential misinterpretation of results and erroneous conclusions. In this study, we systematically evaluated the performance of five TRPV1 and seven TRPA1 commercial antibodies in immunocytochemistry, immunohistochemistry, and western blotting, using both heterologous and native expression systems. We identified two TRPV1 antibodies that consistently yielded robust and specific signals across all techniques and expression models tested; their specificity was validated using a TRPV1 KO mouse. For the remaining antibodies, we provide guidance to facilitate the selection of the most appropriate reagent according to the experimental approach.

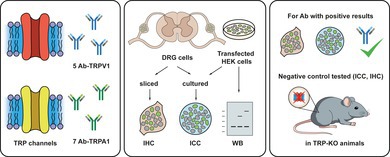

## Graphical abstract

The performance of commercial antibodies against the ion channels TRPV1 and TRPA1 was systematically evaluated, given their key roles in nociception and inflammation. Sensitivity and specificity were assessed using immunocytochemistry, immunohistochemistry, and western blotting in heterologous and native expression systems. Two TRPV1 antibodies showed robust and specific detection, validated in TRPV1 knockout tissue, whereas the remaining antibodies exhibited variable and technique‐dependent performance.

**Abbreviations**
DRG: dorsal root ganglion
ICC: immunocytochemistry
IHC: immunohistochemistry
RRID: Research Resource Identifier
SR: specificity ratio
TRP: transient receptor potential
WB: western blot

## 1 Introduction

Transient receptor potential (TRP) channels are a large, functionally diverse family of ion channels that play central roles in sensory signaling, including the detection of temperature, mechanical, and chemical stimuli. Among the large number of known mammalian TRP proteins (Flockerzi and Nilius 2014), TRPV1 and TRPA1 have received significant attention due to their key involvement in pain and inflammatory processes.

TRPV1 is the archetypal member of the vanilloid TRP subfamily. It is a non‐selective cation channel activated by noxious heat (> 42°C), low pH and various chemical compounds such as capsaicin, the spicy component of chili peppers (Caterina et al. 1997). Because TRPV1 is mainly involved in sensing damaging signals, it is usually referred as a nociceptive ion channel (Bevan et al. 2014). TRPV1 is expressed in several neuronal populations, having particularly high expression in a fraction of primary sensory neurons (Caterina et al. 1997; Sanchez et al. 2001) where it mediates acute noxious temperature sensing as well as thermal hypersensitivity under inflammatory conditions (Caterina et al. 2000; Davis et al. 2000). Outside of nervous system, TRPV1 expression has been identified in multiple tissues, including skin, airways, gastrointestinal tract and immune cells and has been associated with prevalent diseases such as chronic asthma, rheumatoid arthritis and cancer (Bujak et al. 2019).

TRPA1 is also a non‐selective cation channel, in mammals the single member of the ankyrin receptor (TRPA) subfamily. TRPA1 is activated by a plethora of endogenous and exogenous irritants (Talavera et al. 2020; Viana 2016; Vlachova et al. 2024) such as electrophilic compounds, including isothiocyanates, methyl salicylates, cinnamaldehyde and allicin (Hinman et al. 2006; Macpherson et al. 2005), non‐electrophilic compounds, intracellular calcium (Wang et al. 2008; Zurborg et al. 2007), oxygen and oxidative stress (Andersson et al. 2008; Takahashi et al. 2011). TRPA1 activity is modulated by cold (Story et al. 2003) and heat (Vandewauw et al. 2018), however the underlying gating mechanism is not completely understood (Talavera et al. 2020). Whether TRPA1 is mechanosensitive or not is controversial (Moparthi and Zygmunt 2020; Nikolaev et al. 2019). Expression of this protein as an ion channel was initially described in noxious cold‐responding sensory neurons (Story et al. 2003) however, it is also expressed in many non‐neuronal cells including keratinocytes, fibroblasts, endothelial cells, odontoblasts and urothelium (Viana 2016). TRPA1 is a sensor of cellular stress (oxidative stress, hypoxia, noxious temperature), inflammation (Nassini et al. 2014) and tissue damage, including harmful bacterial and viral products (Meseguer et al. 2014).

Both TRPV1 and TRPA1 are of significant interest in the context of pain and inflammation, as their activation is closely associated with the release of pro‐inflammatory substances and the development of chronic pain conditions. They are also involved in pruritus (Mahmoud et al. 2022). Therefore, both channels are potential targets for therapeutic interventions aimed at relieving pain (Levine and Alessandri‐Haber 2007; Marcotti et al. 2023; Mobasheri et al. 2024; Szolcsányi and Sándor 2012; Trevisan et al. 2013). For those reasons, these channels are currently the focus of extensive research. As with other ion channels, there is a plethora of techniques appropriate for their study, including electrophysiology and calcium imaging to examine their function, and RNA in situ hybridization and sequencing, as well as some reporter transgenic mouse lines (Yarmolinsky et al. 2016) to study their expression. While these techniques provide crucial information, detecting the actual protein expression is informative but remains challenging. Often, RNA levels do not correlate with protein membrane expression due to a strong modulation of their trafficking (Marcotti et al. 2023; Schmidt et al. 2009), and the available reporter murine models only reflect transcriptional activity. Thus, antibody‐based techniques such as immunofluorescence and western blot (WB) remain the preferred option to assess protein expression.

Immunocytochemistry (ICC), immunohistochemistry (IHC), and WB are widely used techniques for detection and quantification of proteins. However, reliable commercial antibodies against ion channels, particularly against TRP channels, are scarce and usually require fine‐tuning of their application protocol and thorough characterization and validation (Hernández‐Ortego et al. 2022; Jin et al. 2024; Virk et al. 2019). The use of antibodies without proper controls is particularly problematic and can lead to misinterpretation regarding expression. Here, we characterized five TRPV1 and seven TRPA1 commercial antibodies for their application in WB, ICC, and IHC following common protocols (see Section 2). Antibodies were tested for the detection of heterologous overexpression of TRPV1 and TRPA1 using transfected HEK‐293 cells that were either fixed for ICC or lysed for WB. Those that successfully detected heterologously expressed channels were further tested for native channel expression using dorsal root ganglion (DRG) cultured sensory neurons and DRG sections. Specificity of the antibodies that yielded the best results in DRG‐ICC and/or IHC was confirmed and validated using a TRPV1‐KO mouse.

## 2 Methods

### 2.1 Animals

All experimental procedures were performed in accordance with the Spanish Royal Decree 53/2013 and the European Community Council Directive 2010/63/EU. Adult mice (2–4 months old, 20–40 g weight) of either sex were used. Mice were housed in a temperature‐controlled room (21°C) on a 12 h light/dark cycle, with access to food and water ad libitum. 3–6 animals were housed per cage (conventional polycarbonate, max. capacity: 6 mice).

A total of 12 mice were used: four TRPV1‐EGFP, four TRPV1‐KO, and four TRPA1‐Cre‐ChR2‐YFP. Each immunostaining was performed on four sections or culture coverslips obtained from two different mice. No animals were pooled in the analysis.

### 2.2 Mouse Lines

TRPV1‐EGFP transgenic line from the GENSAT collection, gene: TRPV1‐EGFP (Tg(Trpv1‐EGFP)MA208Gsat/Mmucd, RRID:MMRRC_033029‐UCD), obtained from the MMRRC Repository (033029‐UCD, http://www.informatics.jax.org/allele/MGI:4847511; MMRRC Services, The Rockefeller University, New York, NY). TRPA1‐Cre‐ChR2‐EYFP), generated by crossing TRPA1‐DTR‐Cre (Trpa1tm1(Hbegf, cre)Csz, RRID:MGI:7446805 (Yarmolinsky et al. 2016) with R26 ChR2‐EYFP (B6.Cg‐Gt(ROSA)26Sortm32(CAG‐COP4*H134R/EYFP)Hze/J, JAX stock #024109 (Madisen et al. 2012)). TRPV1 knock out line (B6.129X1‐Trpv1tm1Jul/J, JAX stock #003770. RRID:IMSR_JAX:003770 (Caterina et al. 2000)). The genotype of all mice was confirmed by PCR.

### 2.3 Cell Line Culture and Transfection

The HEK‐293 cell line (not listed by ICLAC) was obtained from Cytion (Heidelberg, Germany), (RRID: CVCL_0045. Lot: 300192‐060922, authenticated on 5.19.2025). Cells were cultured at 37°C, 5% CO_2_ in Dulbecco's Modified Eagle Medium (DMEM, Cat# 61965026, Lot# 2462392) (Thermo Fisher Scientific, Waltham, MA, USA) supplemented with 10% fetal bovine serum (Fetal Bovine Serum, Thermo Fisher Scientific, Cat# 10270106, Lot# 2748891) and 1% penicillin/streptomycin. A frozen stock was prepared after receipt following ECACC guidelines, no further freezing nor authentication was made, cells were used between passages 3–15. Twenty‐four hours prior to transfection, cells were plated in 6‐well culture plates. For ICC, poly‐L‐lysine (0.01%, Sigma‐Aldrich, St. Louis, MO, USA, Cat# P4832‐50ML, Lot# SLCJ7890) was used to coat 6 mm diameter glass coverslips. Cells were transfected with rTRPV1‐EYFP (kindly provided by Antonio Ferrer) or hTRPA1‐tGFP (Sura et al. 2012) using Lipofectamine 2000 reagent (Thermo Fisher Scientific, Cat# 11668019, Lot# 2579994). Each well containing 700 μL DMEM received a transfection mix consisting of 3 μg DNA and 3 μL Lipofectamine in 300 μL Opti‐MEM (Thermo Fisher Scientific, Cat. No. 31985070). After 24–48 h post‐transfection, rTRPV1‐EYFP or hTRPA1‐tGFFP expression was assessed under an epifluorescence microscope, and cells were either lysed (for WB) or fixed in 4% PFA (for ICC).

### 2.4 Mouse DRG Extraction and Neuronal Culture

Mouse DRG neurons were extracted and dissociated as previously described (Hernández‐Ortego et al. 2022). Briefly, the mice were euthanized by cervical dislocation following standard procedures. The spinal cord was removed, and between 20 and 40 DRGs were dissected and rinsed in cold Hank's Balanced Salt Solution (HBSS, Thermo Fisher Scientific (Gibco), Cat# 14170088, Lot# 2700543). For IHC, whole ganglia were immediately fixed for 2 h in 4% PFA. For ICC, ganglia were firstly incubated in a digestion solution containing 900 U/mL type XI collagenase (Collagenase from *Clostridium histolyticum* , Type XI, Sigma‐Aldrich, Cat# C7657‐100MG, Lot# SLCL4567) and 5.46 U/mL dispase (Dispase II (Powder), Gibco (Thermo Fisher Scientific), Cat. No. 11510536, Lot 2440992) for 45 min at 37°C in 5% CO_2_. After enzymatic treatment, the ganglia were mechanically dissociated using fire‐polished glass pipettes in a calcium‐free medium composed of HBSS (Thermo Fisher Scientific), 1% MEM‐Vit (MEM Vitamin Solution (100X), Gibco (Thermo Fisher Scientific), Cat. No. 11120037, Lot 2530989), 10% fetal bovine serum (Thermo Fisher Scientific), and 100 mg/mL penicillin/streptomycin. The cell suspension was centrifuged, and the pellet was resuspended in neuronal culture medium containing MEM (Thermo Fisher Scientific), 1% MEM‐Vit (Thermo Fisher Scientific), 10% fetal bovine serum (Thermo Fisher Scientific), and 100 mg/mL penicillin/streptomycin. The cells were then plated onto 6 mm diameter glass coverslips pre‐coated with 0.01% poly‐L‐lysine (Sigma‐Aldrich, St. Louis). Twenty‐four hours post‐seeding, cells were either subjected to calcium imaging or fixed for 10 min in 4% PFA.

### 2.5 Calcium Microfluometry

The calcium‐sensitive dye Fura‐2 (Thermo Fisher Scientific) was used for ratiometric calcium imaging experiments. The cells were incubated with 5 μM Fura‐2 AM (Invitrogen (Thermo Fisher Scientific), Cat. No. F1221, Lot 2339903) and 400 ng/mL Pluronic F‐127 (Sigma‐Aldrich (Merck), Cat. No. P‐6867, Lot SLCM2345) in a standard external solution containing (in mM) 140 NaCl, 3 KCl, 1 CaCl_2_, 2 MgCl_2_, 10 glucose, and 10 HEPES, at pH 7.2 (300 mOsm/kg), for 45 min at 37°C in 5% CO_2_. After washing with external solution, the coverslips containing the cells were placed in a low‐volume recording chamber mounted on an inverted microscope (Leica DMI3000B, Leica Microsystems, Wetzlar, Germany) and continuously perfused with external solution at a rate of ~1 mL/min. Excitation of Fura‐2 was achieved at 340 and 380 nm using a Lambda 10–2 filter wheel and a Lambda LS xenon arc lamp (Sutter Instruments, Novato, CA, USA). Emitted fluorescence was collected through a 510 nm long‐pass filter. Images were acquired with an Orca ER CCD camera (Hamamatsu Photonics, Hamamatsu, Japan) at a frequency of 0.33 Hz and analyzed using MetaFluor software (Molecular Devices, San Jose, CA, USA). Changes in cytosolic calcium levels are presented as the ratio of emission intensities following sequential excitation at 340 and 380 nm (F340/F380). Temperature‐controlled measurements of HEK‐293 cells and DRG neurons were conducted at 32°C–34°C using a custom‐built water‐cooled Peltier system regulated by a temperature feedback device.

### 2.6 Western Blot

All WB experiments were conducted on samples obtained from HEK‐293 cells transfected with either rTRPV1‐EYFP or hTRPA1‐tGFFP constructs. The cells were collected 48 h post‐transfection, centrifuged at 800×*g* for 10 min, and washed twice with cold PBS. The pellets were lysed using Lysis Buffer (50 mM Tris–HCl, pH 7.5, 120 mM NaCl, 0.5 mM EDTA, 0.5% Nonidet P‐40) supplemented with phosphatase and protease inhibitors (Complete Mini, Roche). The lysates were sonicated for 10 min on ice and centrifuged at 10000×*g* for 15 min at 4°C. Protein concentration was determined using the Pierce BCA Protein Assay Kit (Thermo Fisher Scientific, Cat. No. 23225, Lot VI307953), and samples were mixed with loading buffer (10% sodium dodecyl sulfate, 312.5 mM Tris–HCl pH 6.8, 50% glycerol, and 0.05% bromophenol blue). The protein samples (15 μg per lane) were resolved by 7.5% SDS‐PAGE and transferred onto Protran Nitrocellulose Membrane (Whatman, GE Healthcare Life Science, Chicago, IL, USA, Cat. No. 10401197). Membranes were blocked using 5% powdered milk in TBST (Tris‐buffered saline with 0.05% Tween‐20) and incubated overnight at 4°C with anti‐TRPV1 or anti‐TRPA1 primary antibodies (see Table 1). After washing with TBST, membranes were incubated with an HRP‐conjugated secondary antibody, developed with ECL Plus (GE Healthcare (Cytiva), Cat. No. RPN2132, Lot), and visualized using an Amersham Imager 680 system. Following several TBST washes, membranes were incubated overnight at 4°C with anti‐GFP (1:1000) or anti‐tGFP (1:000), and anti‐GAPDH (1:5000) antibodies, developed, and imaged as described above.

**TABLE 1:** Antibodies used in this study. Identity refers to the percentage of identical residues in the sequence of the immunogen (when available) versus the mouse orthologue.

Name in this study	Target	Species	Company	Immunogen clonality	Identity (vs. mouse)	References
Aviva	TRPA1	Rabbit	Aviva Systems Biology	Human TRPA1 middle region. PC		ARP35205‐P050 RRID:AB_1064077
Novus	TRPA1	Rabbit	Novus Biologicals (Biotechne)	Human TRPA1 N‐terminus (1–100) PC	77%	NB110‐40763 RRID:AB_715124
Millipore A1	TRPA1	Rabbit	Merck Millipore	Human TRPA1 cytoplasmic domain PC		ABN1009 RRID:N/A
Alomone	TRPA1	Rabbit	Alomone Labs	Human TRPA1 747–760. PC	69.2%	ACC‐037 RRID:AB_2040232
Sigma WH	TRPA1	Mouse	Merck Millipore	Human TRPA1 C‐terminus (1033–1117) MC	88.1%	WH0008989M3 RRID:AB_1844053
Santa Cruz A1	TRPA1	Mouse	Santa Cruz Biotechnology	Human TRPA1 C‐terminus (965–1119) MC	85.7%	SC‐376495 RRID:N/A
Proteintech	TRPA1	Rabbit	Proteintech	Human PC		19 124–1‐AP RRID:AB_10642143
Santa Cruz V1	TRPV1	Goat	Santa Cruz Biotechnology	Human TRPV1 C‐terminus PC		SC‐12503 RRID:AB_2209139
Abcam	TRPV1	Rabbit	Abcam	Rat TRPV1 C‐terminus PC		AB6166 RRID:AB_305334
Millipore V1	TRPV1	Rabbit	Merck Millipore	Rat TRPV1 C‐terminus PC		AB5370P RRID:AB_91816
Neuromics GP	TRPV1	Guinea Pig	Neuromics	Rat TRPV1 C‐terminus (824–838) PC	92.8%	GP14100 RRID:AB_1624142
Neuromics GT	TRPV1	Goat	Neuromics	Rat TRPV1 4–21 PC	80.9%	GT15129 RRID:AB_2209002
EYFP	GFP/YFP	Chicken	Abcam	Isolated from jellyfish *Aequorea victoria* GFP PC		ab13970 RRID:AB_300798
anti tGFP	GFP	Chicken	Origene	Purified tGFP protein expressed in *E. coli* PC		TA150075 RRID:N/A
anti β‐tub	Beta‐III‐tubulin	Rabbit	Biolegend	Rat brain microtubules PC		802 001 RRID:AB_2564645
anti β‐tub	Beta‐III‐tubulin	Mouse	Biolegend	Rat brain microtubules PC		801 201 RRID:AB_2313773
EGFP	GFP/YFP	Rabbit	Invitrogen	Isolated from jellyfish *Aequorea victoria* GFP PC		A6455 RRID:AB_2314549
GAPDH	GAPDH	Rabbit	Sigma	Synthetic peptide: residues 314–333 of mouse GAPDH		G9545 RRID:AB_796208
anti tGFP	GFP	Goat	Origene	Purified tGFP protein expressed in *E. coli* PC		TA150096 RRID:N/A
Mouse HRP	Mouse	Rabbit	Sigma	Rabbit IgG fraction antiserum		A9044 RRID:AB_258431
Rabbit HRP	Rabbit	Goat	Sigma	Goat IgG fraction antiserum		A9169 RRID:AB_258431
Goat anti‐chicken IgY 488	Chicken	Goat	Molecular Probes Invitrogen	Chicken gamma globulin heavy and light chains PC		A11039//1 458 638 RRID:AB_142924
Goat anti‐Mouse IgG (H + L) 647	Mouse	Goat	Molecular Probes Invitrogen	Mouse gamma globulin heavy and light chains PC		A21237 RRID:AB_1500743
Donkey anti‐Rabbit 555	Rabbit	Donkey	Abcam	N/A. PC		ab150062 RRID:AB_2801638
Anti‐Guinea Pig 647	Guinea Pig	Goat	Molecular Probes Invitrogen	Guinea pig gamma globulin heavy and light chains PC		A21450 RRID:AB_2535867
Anti‐Goat 647	Goat	Donkey	Invitrogen	Goat gamma globulin heavy and light chains PC		AB_2535864 RRID:AB_2535864

Abbreviations: ICC, immunocytochemistry; IHC, immunohistochemistry; MC, monoclonal; PC, polyclonal. WB, western blotting.

### 2.7 Immunocytochemistry

Cells cultured on glass coverslips were fixed in 4% PFA (10 min), followed by three washes in PBS and two washes in TTBS (0.5 M Tris Base, 9% w/v NaCl, 0.5% Tween‐20, pH 7.6) for 10 min. To block nonspecific binding, cells were incubated for 30 min in freshly prepared blocking solution containing 1X TTBS, 1% bovine serum albumin (Tocris Bioscience, Bristol, UK, Cat. No. 5217), and 0.25% Triton‐X100.

Next, cells were incubated for 2 h at room temperature (RT) with the primary antibodies, followed by three 10 min washes with TTBS. Then, they were incubated for 45 min at RT with the secondary antibodies, both diluted in blocking solution. After another round of TTBS washes (three times), an additional step was performed for HEK cells, which were incubated for 5 min with Hoechst 33342 (Thermo Fisher Scientific, Cat. No. H3570; RRID:AB_10626776) to stain the nuclei.

Finally, cells were washed with PBS, rinsed once with ddH_2_O, and mounted on microscope slides using VectaShield H‐1000 antifade mounting medium (Vector Laboratories, Burlingame, CA, USA, RRID:AB_2336789). Coverslips were sealed with clear nail polish and stored at 4°C until imaging.

Z‐stack images (step size: ~2 μm) were acquired using either a UPlanSApo 20× objective on an Olympus FV1200 inverted confocal microscope, controlled by FV10‐ASW 4.2 software (Olympus Life Sciences, Waltham, MA, USA, RRID:SCR_014215). Identical acquisition settings (laser power, gain, and offset) were used for all fluorescence images.

### 2.8 Immunohistochemistry

Fresh whole dorsal root ganglia (DRGs) were fixed in 4% PFA (1 h), washed twice in PBS (10 min each), and cryoprotected overnight at 4°C in 30% sucrose. The next day, the tissue was embedded in OCT Tissue‐Tek (Sakura Finetek, Torrance, CA, USA, Cat. No. 4583 Lot 2303909830) and frozen in dry ice. Tissue sections (20 μm thick) were obtained using an MNT cryostat (Slee Medical, Nieder‐Olm, Germany) and mounted onto SuperFrost microscope slides (Thermo Fisher Scientific). The slides were dried at 37°C for 30 min and washed twice with PBT (0.1 M PB, 0.05% Tween‐20, pH 7.4). Non‐specific binding sites were blocked for 1 h in a solution containing 5% bovine serum albumin and 1% Triton X‐100 in PBT. Slides were incubated overnight at 4°C with primary antibodies, followed by four 10 min washes in PBT the next day. Then, the slides were incubated for 2 h at room temperature (RT) with secondary antibodies (both diluted as indicated in Table 1). After incubation, slides were washed four times with PBT (10 min each), once with PBS (10 min), and once with ddH_2_O (5 min). Finally, the slides were dried in dark conditions at RT, and a glass coverslip was mounted using Fluoromount mounting medium (Sigma‐Aldrich, Cat. No. F4680). Z‐stack images (step size: ~2 μm) were acquired using a UPlanSApo 20X on an Olympus FV1200 inverted confocal microscope, controlled by FV10‐ASW 4.2 software (Olympus Life Sciences). Identical acquisition settings (laser power, gain, and offset) were used for all fluorescence images.

### 2.9 Image Analysis and Quantification

All quantitative measurements were performed using 16‐bit raw maximum projection images, without any further modifications. To assess antibody specificity, we defined the specificity ratio (SR) in a TRPV1^+^ cell (defined as a cell labeled with the antiGFP antibody) as the mean signal intensity of the TRPV1 antibody fluorescence in that cell, divided by the average mean signal intensity in several (at least 20) TRPV1^−^ cells in the same field. An SR value of 1 indicates similar fluorescence intensity in TRPV1^+^ and TRPV1^−^ cells, while an SR > 1 indicates higher fluorescence intensity in TRPV1^+^ than in TRPV1^−^ cells. Image analysis was performed using ImageJ 1.51j8 (NIH, Bethesda, MD, USA, RRID:SCR_003070) and Origin 2023b (OriginLab, Northampton, MA, USA, RRID:SCR_014212). The same procedure was applied for evaluation of TRPA1 labeling, comparing TRPA1^+^ cells to TRPA1^−^ cells in each field, when analyzing heterologous channel expression in HEK293 cells. SR was not calculated for endogenous TRPA1 expression in DRG as with TRPA1‐Cre mice, recombination only occurs in a fraction of TRPA1 expressing neurons. The brightness and contrast of the immunofluorescence images were adjusted only for presentation purposes, enhancing visualization without altering any other feature. For the TRPV1 antibody signal images, the maximum red pseudocolor level was set to match the maximum fluorescence intensity. As a result, images displaying high background noise correspond to experiments where the antibody signal was poor. The same procedure was applied for TRPA1 antibody signal images. The same display settings were used for all images of the same antibody and technique.

Quantification was performed by an experimenter blind to the antibody and mouse used.

### 2.10 Statistical Analysis

The values are presented as indicated in each figure caption. The normality of data distribution was assessed using the Shapiro–Wilk and Kolmogorov–Smirnov tests. For antibody dilution comparison, statistical significance was determined using the Mann–Whitney test. A *p*‐value < 0.05 was considered statistically significant. Significance was categorized as *p < 0.05, **p < 0.01 and ***p < 0.001.

The performance of the different antibodies was assessed as the difference in medians relative to the control, using the Hodges–Lehmann estimator. To determine the sample size required to achieve a statistical power of 0.80, a Monte Carlo simulation (*B* = 2000, assuming a lognormal distribution of the data) was conducted in Python 3. The simulation indicated that *n* = 70 per group (1‐β = 0.823) would be needed to detect a median difference of 0.5 units in specificity ratios.

All statistical analyses were conducted using Prism version 8 (GraphPad Software, San Diego, CA, USA, RRID:SCR_002798). A detailed summary of all analyses performed is provided as Supporting Information. No test for outliers was conducted, and no data points were excluded.

## 3 Results

Here, we characterized 12 commercially available antibodies, 5 directed against TRPV1 and 7 against TRPA1. These antibodies were generated against different channel orthologues (human, mouse, or rat), although cross‐species reactivity has been reported for all of them. Among the tested antibodies, four TRPV1 antibodies recognized epitopes within the intracellular C‐terminus and one within the N‐terminus, while TRPA1 antibodies targeted distinct regions of the channel. However, the precise epitope sequences were not disclosed for all antibodies, hindering the assessment of sequence identity across orthologues.

### 3.1 Heterologous rTRPV1 and mTRPA1 Expression in HEK‐293 Cells Was Detected by Most Antibodies Tested

**FIGURE 1:**
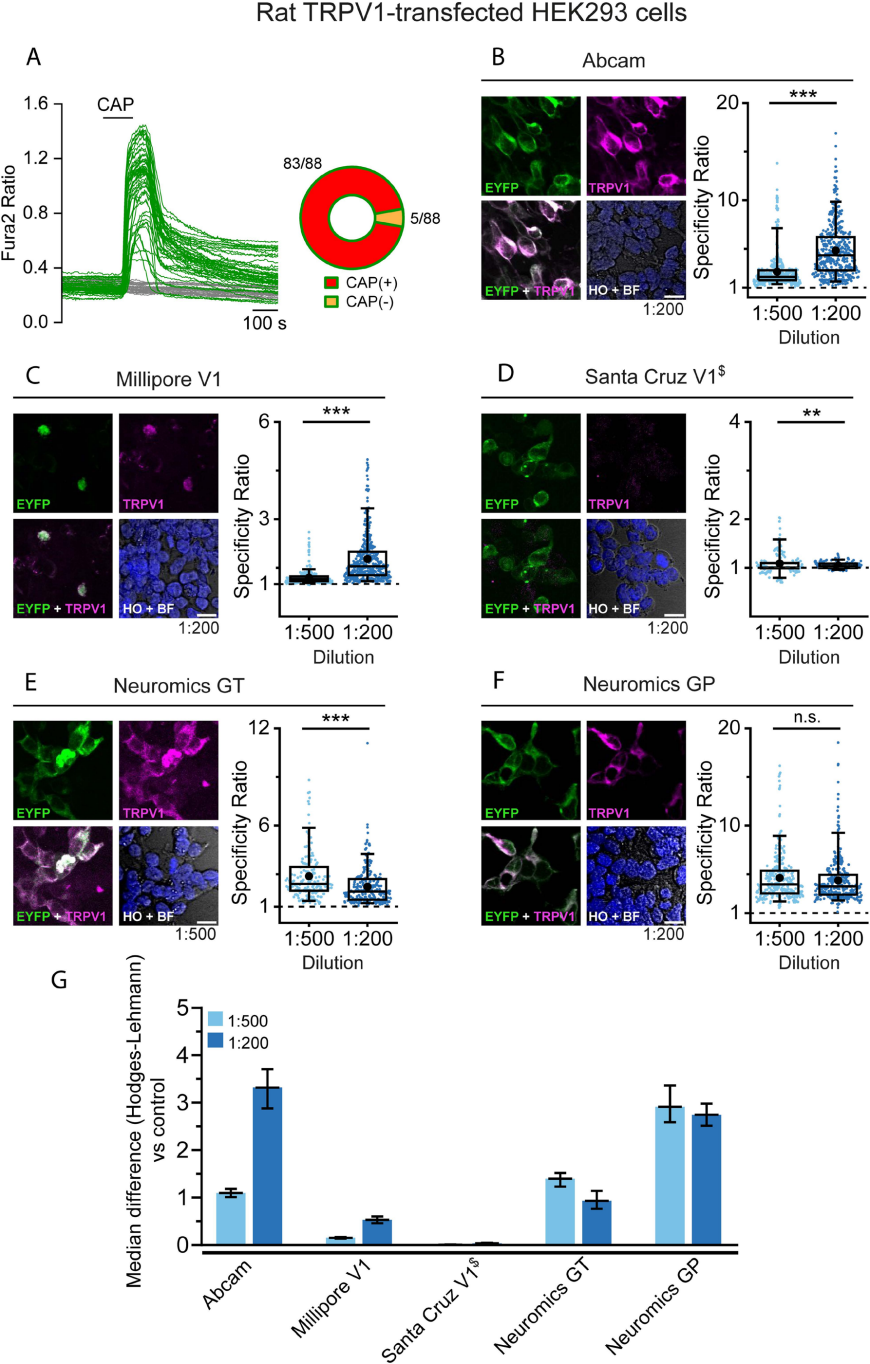
Calcium imaging and immunocytochemistry showing TRPV1 expression in transfected HEK‐293 cells. (A) Fura2 calcium imaging in HEK‐293 cells transiently expressing rTRPV1‐EYFP fusion protein. Representative traces of calcium transients evoked by capsaicin in EYFP^+^ cells. Green traces correspond to EYFP^+^ cells and gray traces to EYFP^−^ cells. Pie chart represents the percentage of EYFP^+^ cells that responded or failded to respond to capsaicin. Data obtained from 88 cells from 3 coverslips. (B–F left) Confocal images of TRPV1‐EYFP transiently expressed in HEK‐293 cells. EYFP (green), TRPV1 antibody (magenta) and Hoechst (HO, blue). Merge images correspond to the overlap between TRPV1 and EYFP on the left and Hoechst and brightfield (BF) on the right. Antibody dilution of the representative images is indicated in the lower right corner. Scale bar: 20 μm. (B–F right) Box plots display the specificity ratio (SR) for each tested dilution of the corresponding antibody. Each dot represents a single EYFP^+^ cell. Boxes indicate the interquartile range (25th to 75th percentiles) and whiskers extend to the 5th and 95th percentiles. The horizontal line inside each box marks the median and the black dot indicates the mean. (***p < 0.001 Mann–Whitney test). (G) Bar histogram graph summarizes the SR median difference between the tested antibody dilution and their controls lacking primary antibody (secondary antibody only; rabbit, goat or guinea pig) as calculated with the Hodges‐Lehman estimator. Error bars represent the 95% confidence interval. For each antibody and dilution, a minimum of 100 cells were analyzed across 4 fields from 2 independent transfections. ^$^Santa Cruz V1 antibody was generated against a human TRPA1 peptide.

**FIGURE 2:**
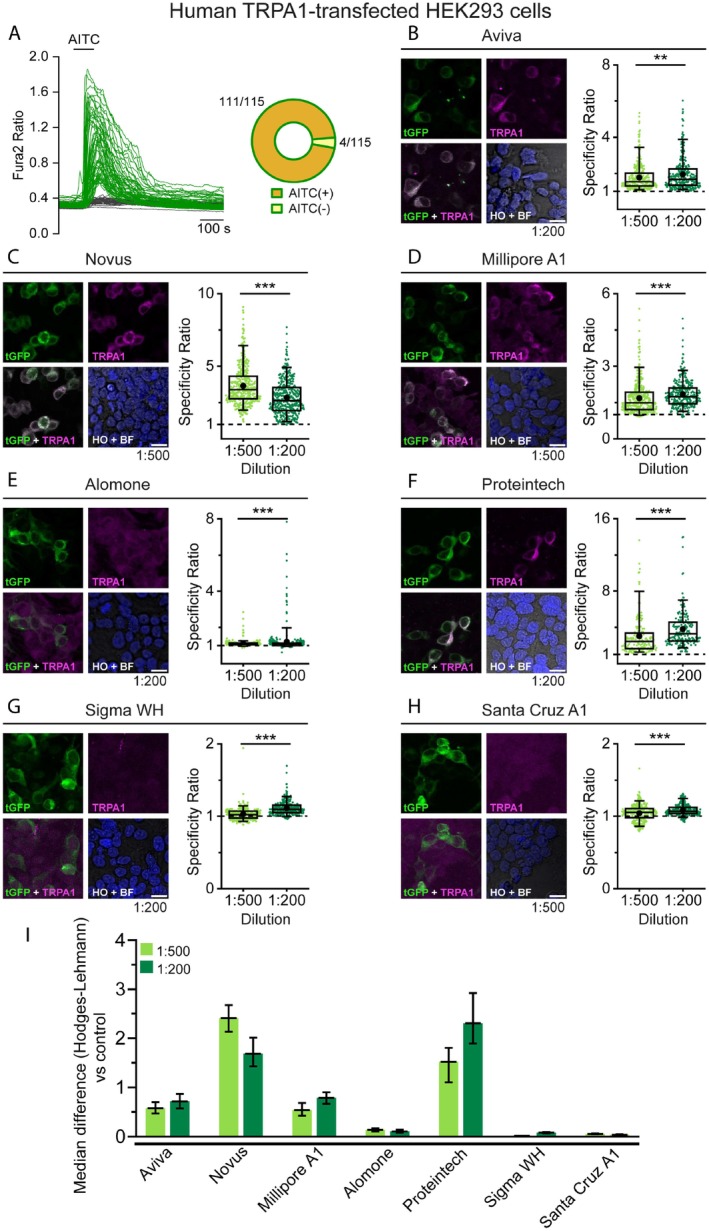
Calcium imaging and immunocytochemistry of TRPA1 expression in transfected HEK‐ 293 cells. (A) Representative traces of Fura2 calcium transients evoked by 50 μM AITC. Green traces correspond to tGFP^+^ cells and gray traces to tGFP^−^ cells. The pie chart represents the proportion of tGFP^+^ cells that responded or failed to respond to AITC. Data obtained from 115 cells from 3 coverslips. (B–H left) Confocal images of TRPA1‐tGFP transiently expressed in HEK‐ 293 cells incubated with the indicated antibody. tGFP (green), TRPA1 (magenta) and Hoechst staining (blue). Merge images correspond to the overlap between TRPA1 and tGFP (lower‐left panel) or Hoechst and brightfield (HO, BF, lower‐right). Antibody dilution of the representative images is indicated in the lower right‐hand corner. Scale bar: 20 μm. (B–H right) box plots display the specificity ratio (SR) for each tested dilution of the corresponding antibody. Each dot represents a single tGFP^+^ cell. Boxes indicate the interquartile range (25th to 75th percentiles) and whiskers extend to the 5th and 95th percentiles. The horizontal line inside each box marks the median and the black dot indicates the mean. (**p < 0.01, ***p < 0.001 Mann–Whitney test). (I) Bar histogram graph summarizes the SR median difference between the tested antibody dilution and their controls lacking primary antibody (secondary antibody only; rabbit or mouse) as calculated with the Hodges‐Lehman estimator. Error bars represent the 95% confidence interval. For each antibody and dilution, a minimum of 319 cells were analyzed across 4 fields from 2 independent transfections.

To assess the efficiency of each antibody in detecting overexpression of the corresponding channel by immunocytochemistry (ICC), we transfected cultured HEK‐293 cells with rTRPV1‐EYFP or hTRPA1‐tGFP fusion proteins. EYFP or tGFP fluorescence served to identify cells expressing the channel. To validate this approach, we first performed calcium imaging and confirmed that fluorescently‐labeled HEK cells responded to specific TRPV1 and TRPA1 agonists. Specifically, 94% of EYFP^+^ (TRPV1‐expressing) cells responded to 100 nM capsaicin, whereas only 6% of EYFP^+^ cells were insensitive to it (Figure 1A). Conversely, in TRPA1 experiments, 97% of tGFP^+^ (TRPA1‐expressing) cells responded to 50 μM AITC and only 3% of tGFP^+^ were not activated (Figure 2A).

Following common vendors' recommendations, we tested two antibody dilutions (1:500 and 1:200). Four TRPV1 antibodies showed clear co‐localization with EYFP signals (Figure 1B,C,E,F, Figure S2A), confirming successful detection of overexpressed rTRPV1. The Santa Cruz V1 antibody showed no detectable activity (Figure 1D). No TRPV1 immunostaining was observed in EYFP^−^ (non‐transfected) cells within the same field, indicating minimal nonspecific staining. To compare antibody performance, we calculated a specificity ratio (SR; see Section 2) for each antibody and dilution (Figure 1B–F, box plots). Interestingly, SR values varied between dilutions (except for the anti‐TRPV1 Neuromics GP antibody). The highest concentration was not always the best, highlighting the need to optimize each antibody dilution for reliable detection.

When SR values from different antibodies and dilutions were compared with the control obtained using only the corresponding secondary antibodies (Figure 1G), 4 of the 5 tested antibodies displayed SR higher than the corresponding control (secondary anti‐rabbit or anti‐goat), indicating stronger fluorescence in TRPV1^+^ than in TRPV1^−^ cells. Among them, the Abcam and Neuromics GP antibodies yielded the highest median difference versus control, suggesting superior performance.

Equivalent experiments were performed with seven antibodies directed against TRPA1. Four of these antibodies showed clear co‐localization of tGFP and TRPA1 (Figure 2B–D,F, Figure S2D), indicating successful detection of overexpressed hTRPA1. The remaining three antibodies failed to show co‐localization between reporter and antibody fluorescence (Figure 2E,G,H). Consistently, the four functional antibodies displayed SR values higher than controls (anti‐rabbit or anti‐mouse) without primary antibodies (Figure 2I). Novus and Proteintech antibodies displayed large median differences compared to the control.

### 3.2 Selective Detection of rTRPV1 and hTRPA1 by Western Blot

**FIGURE 3:**
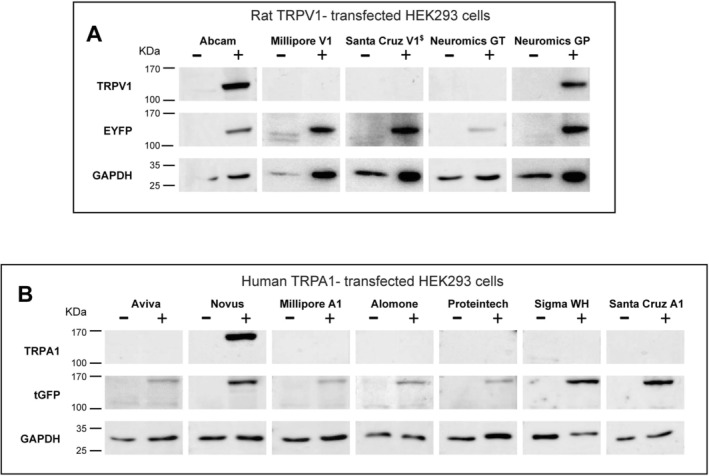
Western blot analysis for TRPV1 and TRPA1 antibodies specificity. (A) Immunoblots for TRPV1 using Abcam (1:1000), Millipore V1 (1:1000), Santa Cruz V1 (1:100), Neuromics GT (1:200), and Neuromics GP (1:1000) antibodies. (−) lanes: Untransfected HEK‐293 cells, (+) lanes: HEK‐293 cells transfected with rTRPV1‐EYFP (B) Immunoblots for TRPA1 using Aviva (1:300), Novus (1:500), Millipore A1 (1:500), Alomone (1:200), Proteintech (1:500), Sigma WH (1:500), and Santa Cruz A1 (1:100). (−) lanes: Untransfected HEK‐293 cells, (+) lanes: HEK‐293 cells transfected with hTRPA1‐tGFFP. For each antibody, top row: Immunoblot with each TRPV1/TRPA1 antibody. Middle row: EYFP/tGFP immunoblotting of the same membrane. Bottom image: GAPDH loading control. Each blot was performed at least three times to rule out technical artifacts in cases where no anti‐TRPV1/TRPA1 signal was detected. For every repetition, the identical lysate sample was probed with all antibodies. Full uncropped blots are available in the Figure S1. ^$^Santa Cruz V1 antibody was generated against a human TRPA1 peptide.

Antibody validation was further assessed by western blot (WB). HEK‐293 cells were transfected with rTRPV1 or hTRPA1, and cell lysates were subsequently analyzed (Figure 3A,B). The rTRPV1‐EYFP fusion protein is expected to migrate at ~125 kDa, while hTRPA1‐tGFP should appear at ~160 kDa. Only the Abcam and Neuromics GP antibodies successfully detected TRPV1 (Figure 3A, full uncropped blots in Figure S1), consistent with their superior efficiency in ICC of TRPV1‐transfected cells (Figure 1G). The remaining three TRPV1 antibodies did not produce a distinguishable band. Notably, probing with anti‐EYFP antibodies revealed consistent bands corresponding to rTRPV1‐EYFP, confirming the presence of the fusion protein in the extracts (Figure 3A, middle row).

Surprisingly, detection of hTRPA1 by WB was more challenging. Of the seven antibodies tested, only the Novus antibody produced a band of the expected size (Figure 3B; full uncropped blots in Figure S1). Unexpectedly, the Aviva and Proteintech antibodies, which performed well in ICC of hTRPA1‐transfected cells, failed to detect a band in WB. As observed with TRPV1, the presence of the fusion protein in all lysates was verified using antibodies against the fluorescent reporter (tGFP).

### 3.3 Three TRPV1 Antibodies Consistently Detect Endogenous TRPV1 in Mouse Dorsal Root Ganglion Neurons

After validating the specificity of the antibodies in heterologous expression systems, we next examined whether those that performed well in ICC of transfected HEK‐293 cells could detect endogenous channel expression in mouse dorsal root ganglion (DRG) sensory neurons, using both cultured neurons ICC and DRG slices IHC.

We used a TRPV1‐EGFP transgenic mouse line to identify TRPV1^+^ neurons. In this reporter line, 96% of fluorescent cells respond to capsaicin, whereas 92% of non‐fluorescent cells are unresponsive, as previously characterized (Fernández‐Trillo et al. 2020).

**FIGURE 4:**
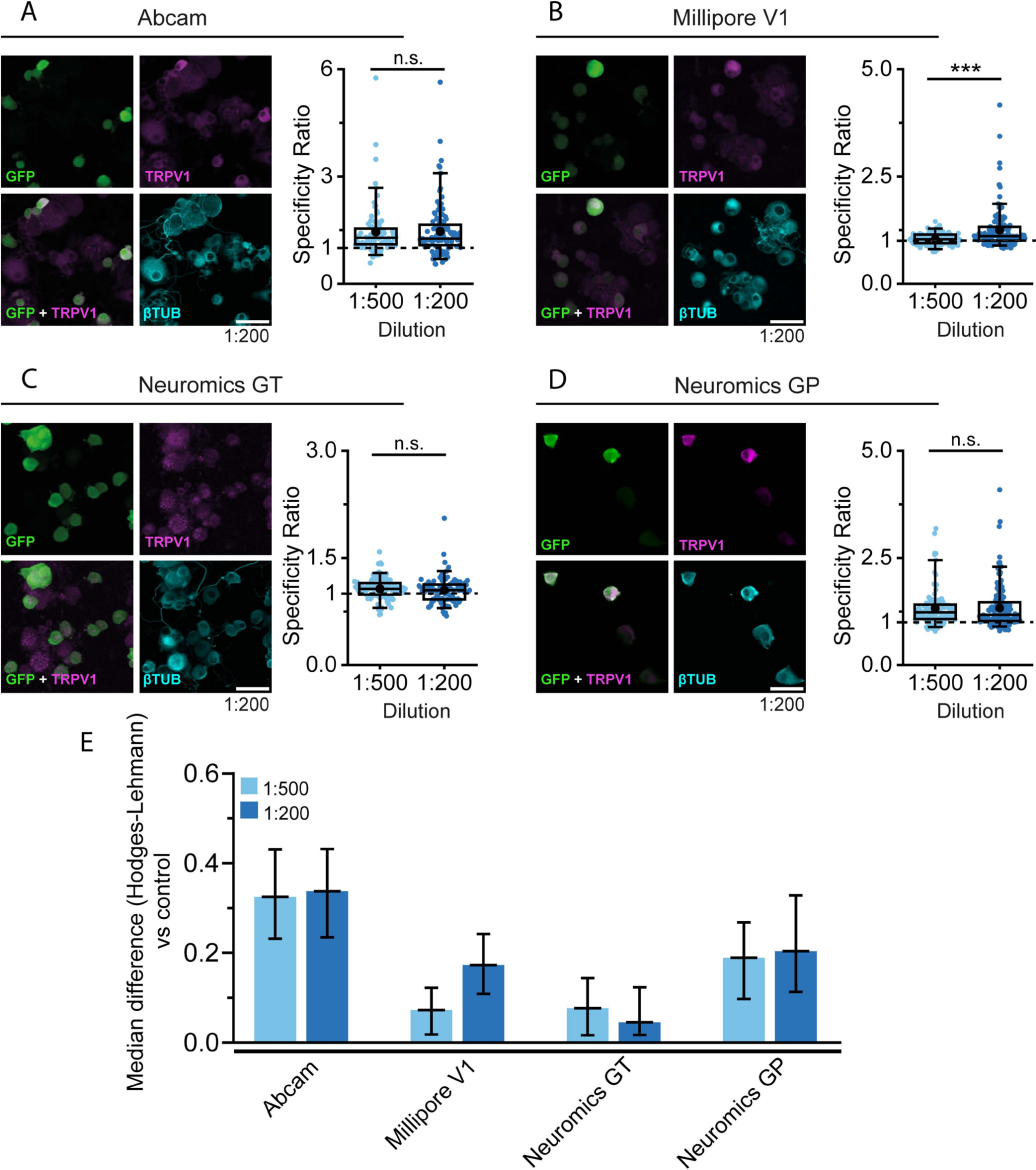
Immunocytochemistry of endogenously expressed TRPV1 in DRG cultures from rTRPV1‐EGFP transgenic mice. (A–D left): Confocal images of TRPV1^+^ DRG neurons incubated with the indicated antibody. EGFP (green), TRPV1 antibody (magenta) and βIII‐Tubulin (cyan). Scale bar: 50 μm. (A–D right) box plots display the specificity ratio (SR) for each tested dilution of the corresponding antibody. Each dot represents a single EGFP^+^ cell. Boxes indicate the interquartile range (25th to 75th percentiles) and whiskers extend to the 5th and 95th percentiles. The horizontal line inside each box marks the median and the black dot indicates the mean. (***p < 0.001 Mann–Whitney test). (E) Bar histogram graph summarizes the SR median difference between the tested antibody dilution and their controls lacking primary antibody (secondary antibody only; rabbit, goat or guinea pig) as calculated with the Hodges‐Lehman estimator. Error bars represent the 95% confidence interval. For each antibody and dilution, a minimum of 92 cells were analyzed across 4 microscopic fields from 2 different animals.

We first performed ICC for TRPV1 in cultured DRG neurons, using the same antibody dilutions (1:200 and 1:500) as in HEK‐293 ICC. Antibodies from Abcam, Millipore V1, Neuromics GT, and Neuromics GP were tested (Figure 4A–D, Figure S2B). In all cases, some nonspecific staining was observed in TRPV1^−^ cells. Nevertheless, fluorescence intensity in TRPV1^+^ neurons was consistently higher with Abcam, Millipore (1:200), and Neuromics GP antibodies, indicating their ability to detect the endogenous channel. As expected, labeled neurons were predominantly small to medium in size, consistent with known TRPV1 expression in nociceptors. Although SR values were generally low (Figure 4A,B,D), these three antibodies yielded small but detectable SR median differences versus the no‐primary antibody controls (Figure 4E). The only exception was the Millipore V1 antibody, which appeared to require a higher concentration, as at a 1:500 dilution the median difference versus control is particularly small. For Abcam and Neuromics GP antibodies, SR values did not vary with dilution, suggesting that their performance is less dependent on antibody concentration.

Antibodies showing strong performance in ICC were further evaluated in tissue sections using IHC. Antigen retrieval with citrate treatment was not applied because our preliminary tests showed no improvement. Consistently, our previous study evaluating TRPM8 antibodies also reported that citrate treatment was ineffective (Hernández‐Ortego et al. 2022).

**FIGURE 5:**
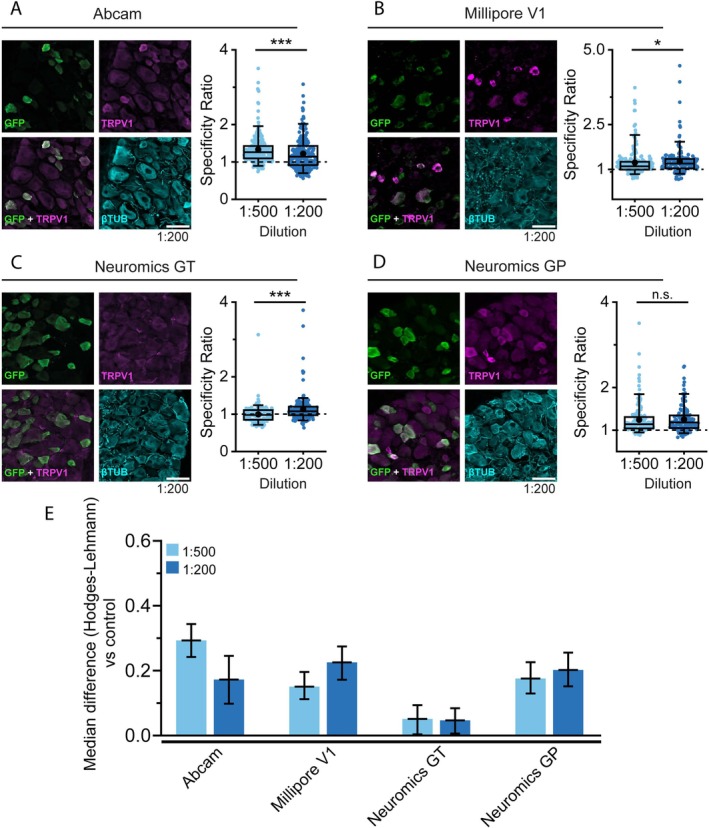
Immunohistochemistry of endogenously expressed TRPV1 in DRG slices from rTRPV1‐ EGFP transgenic mice. (A–D left): Confocal images of TRPV1^+^ DRG neurons incubated with the indicated antibody. EGFP (green), TRPV1 antibody (magenta) and βIII‐Tubulin (cyan). Scale bar: 50 μm. (A–D right) box plots display the specificity ratio (SR) for each tested dilution of the corresponding antibody. Each dot represents a single EGFP^+^ cell. Boxes indicate the interquartile range (25th to 75th percentiles) and whiskers extend to the 5th and 95th percentiles. The horizontal line inside each box marks the median and the black dot indicates the mean. (*p < 0.05, ***p < 0.001 Mann–Whitney test). (E) Bar histogram graph summarizes the SR median difference between the tested antibody dilution and their controls lacking primary antibody (secondary antibody only; rabbit, goat or guinea pig) as calculated with the Hodges‐Lehman estimator. Error bars represent the 95% confidence interval. For each antibody and dilution, a minimum of 141 cells were analyzed across 4 pictures from 2 different animals.

IHC results (Figure 5A–D, Figure S2C) were consistent with those obtained by ICC (Figure 4A–D). The Abcam, Millipore V1, and Neuromics GP antibodies gave the best results, showing stronger fluorescence in TRPV1^+^ than in TRPV1^−^ cells (Figure 5A,B,D). However, all three also produced noticeable nonspecific staining. Both dilutions of the three functional antibodies yielded SR values higher than their respective controls, albeit the median difference is small, suggesting that these antibodies could be suitable for detecting endogenous TRPV1, provided that the IHC protocol is further optimized. Notably, the dilution of the Abcam and Millipore V1 antibodies may further improve the results (Figure 5A,B). In contrast, the Neuromics GT antibody failed to label TRPV1^+^ neurons (Figure 5C,E).

### 3.4 The Specificity of the Best TRPV1 Antibodies Was Validated in TRPV1‐KO Mice

**FIGURE 6:**
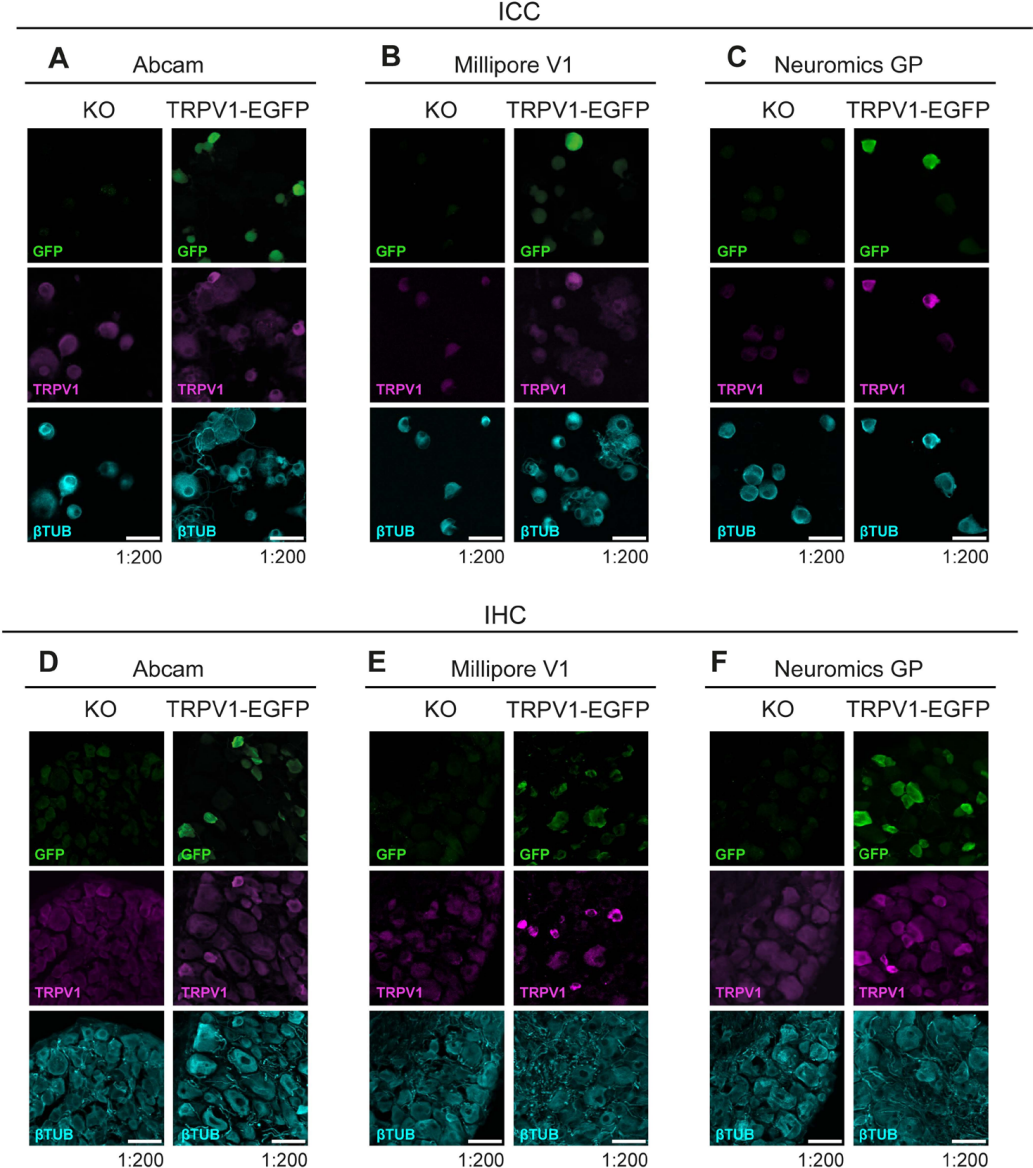
Comparison of immunofluorescence of endogenously expressed TRPV1 in cultured cells and slices from TRPV1‐KO (KO) and TRPV1‐EGFP mice. (A–C) Immunocytochemistry. (D–F) Immunohistochemistry. (A–) Confocal images of cultured DRG neurons and DRG tissue sections (D–F) from TRPV1‐KO (KO, left) and TRPV1‐EGFP (right). EGFP (green), TRPV1 antibody (magenta), and βIII‐Tubulin (cyan). Scale bar: 50 μm. (A–F) For each antibody and dilution, 4 pictures from 2 different animals were studied, image display settings are the same for KO and TRPV1‐EGFP.

The current gold standard for antibody validation is their testing in KO models for the protein/antigen target of interest (Bordeaux et al. 2010). Accordingly, we assessed the specificity of the Abcam, Millipore V1, and Neuromics GP antibodies by performing ICC and IHC in DRG cultured neurons and slices from TRPV1 KO mice. Note that neither TRPV1 mRNA nor protein is detected in this KO mouse line (Caterina et al. 2000). As expected, none of the three antibodies produced a distinguishable signal above the nonspecific background fluorescence observed in all cells, confirming that the labelling seen in TRPV1‐EGFP mice reflects specific detection of the channel (Figure 6). Enhanced nonspecific fluorescence visualization is provided in Figure S3. Since the KO line lacks a fluorescent reporter, SR values could not be calculated; as an alternative, the fluorescence variance was used to assess the differential staining in TRPV1‐EGFP and TRPV1‐KO mice (Figure S4). Nevertheless, anti‐GFP staining was used as an additional control, confirming that all cells displayed comparable nonspecific fluorescence levels unlike what was observed with TRPV1‐EGP animals.

### 3.5 None of the Commercial TRPA1 Antibodies Tested Showed Specific Staining in Native Tissues

**FIGURE 7:**
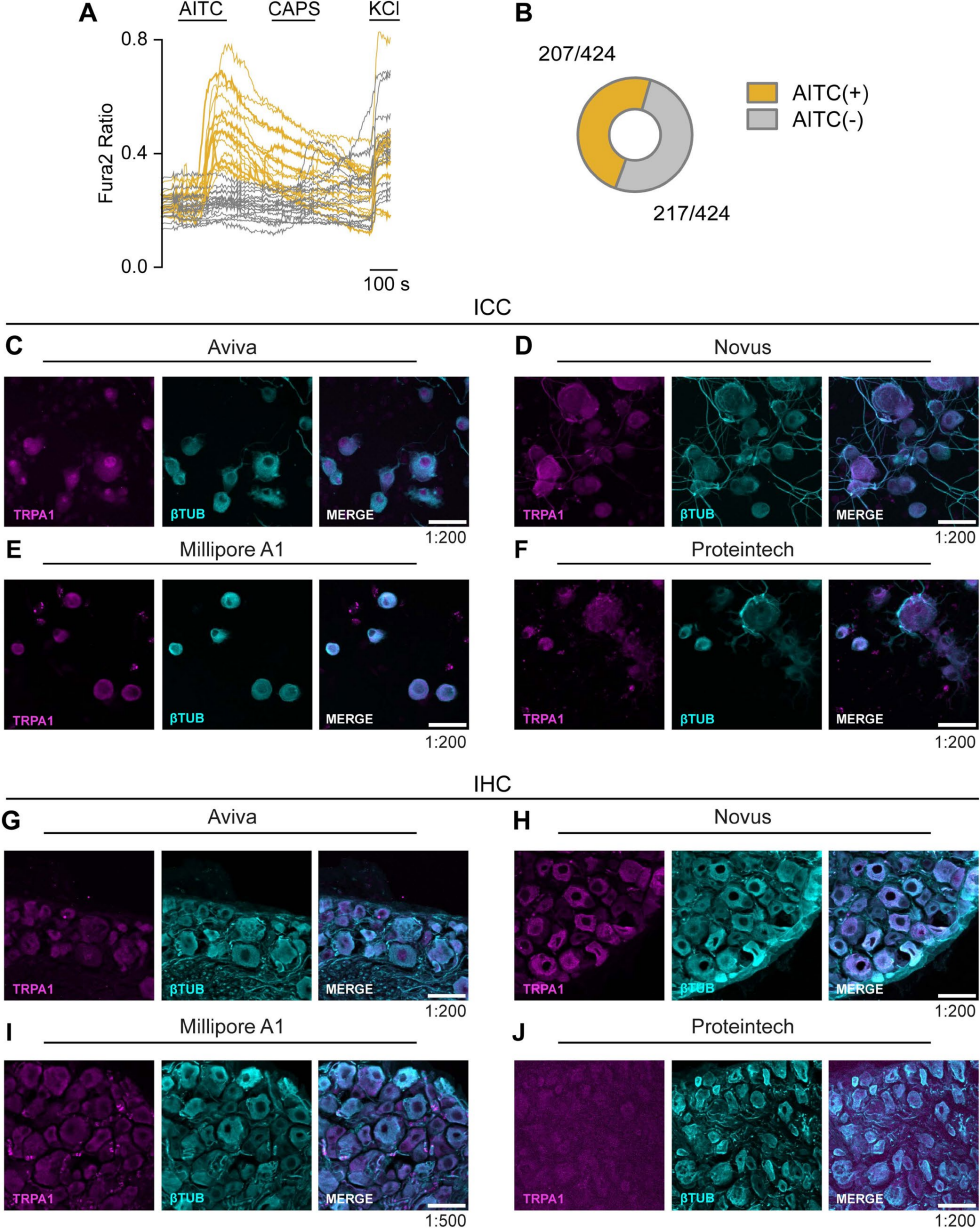
Calcium imaging and Immunochemistry of endogenous TRPA1 in cultured DRG neurons from TRPA1‐Cre‐ChR2‐EYFP mice. (A) Representative traces of calcium transients evoked by AITC and capsaicin in DRG neurons. Traces corresponding to cells responding to 50 μM AITC are displayed in yellow and non‐responding cells are displayed in gray. (B) The pie chart represents the proportion of neurons responding or failing to respond to AITC. Data obtained from 424 cells from 5 coverslips. (C–F) ICC or (G–J) IHC confocal images of DRG sensory neurons incubated with the indicated antibody. TRPA1 antibody (magenta) and βIIITubulin (cyan), merge images display TRPA1 antibody signal + βIII‐Tubulin. Dilution of the corresponding antibody shown in each example is indicated in the lower right corner. Scale bar: 50 μm. Representative images were chosen from 4 pictures of 2 different animals.

We also performed ICC and IHC in cultured mouse DRG neurons and tissue slices to validate TRPA1 antibodies. For these experiments, we used the TRPA1‐Cre‐ChR2‐EYFP transgenic mouse line, expressing EYFP in TRPA1^+^ neurons. Using calcium imaging, we observed that although 96% of EYFP^+^ neurons responded to the TRPA1 agonist AITC, 43% of EYFP^−^ neurons also responded, indicating specific but inefficient recombination (Figure S5). For this reason, we could not calculate SR values. Since a reliable reporter was not available, we used calcium imaging to estimate the fraction of total DRG neurons responding to AITC 50 μM (i.e., TRPA1‐expressing neurons). We found that approximately 50% of neurons were TRPA1^+^ (Figure 7A,B), therefore, a reliable TRPA1 antibody would be expected to selectively label roughly half of the neuronal population. We tested the four antibodies that performed well in ICC of TRPA1‐transfected HEK‐293 cells, but none produced specific staining. In all cases, all neurons identified by β‐III‐tubulin showed uniform staining (Figure 7C–J). Taken together, our results indicate that, under the conditions tested, the four antibodies can detect heterologous TRPA1 expression in transfected cells but fail to recognize endogenous TRPA1 in mouse DRG neurons or tissue.

## 4 Discussion

Antibody‐based methods are among the most widely used and informative approaches in cell biology to assess protein abundance and distribution. However, it is well recognized that many antibodies perform poorly, often displaying limited sensitivity and lacking specificity (Bordeaux et al. 2010). Very often, negative results remain unpublished, and different research groups will stumble against the same problem, unaware of the poor performance of a particular reagent. In addition, in many studies, antibody specificity is not thoroughly evaluated, which may lead to misinterpretation of the results, limiting research reproducibility. In the case of antibodies used as biomarkers, an uncritical evaluation could lead to misdiagnosis or faulty patient stratification (Howat et al. 2014). For these reasons, rigorous validation and characterization of antibodies is of critical importance (Bordeaux et al. 2010).

Transient receptor potential (TRP) channels have important physiological roles (Bevan et al. 2014). Like most membrane proteins, their expression is generally lower than for cytoplasmic proteins. Since immunodetection of TRP channels is particularly challenging, we compared the performance of several commercially available antibodies against TRPV1 and TRPA1 across commonly used techniques (WB, ICC, and IHC). To enhance the power of the validation, all the antibodies were evaluated under identical experimental conditions.

Selecting antibodies from the wide range of commercially available options remains challenging, as to our knowledge, no universally accepted scientific criteria currently guide this choice, beyond excluding reagents with previously reported poor or questionable performance. Although the species origin of the immunogen relative to the target may be considered an important factor, this criterion is not definitive and likely depends more on the degree of sequence identity between the immunogen and the target, which is often highly conserved but not always disclosed by manufacturers. For example, non‐commercial TRPA1 antibodies generated against human immunogens have been successfully validated in TRPA1 knockout models for detecting mouse TRPA1 (Stokes et al. 2006; Uchiyama et al. 2020). A pragmatic selection strategy was therefore applied, based on published literature, vendor specifications, and recommendations from colleagues. Notably, other antibody validation studies similarly lack explicit selection criteria (Birgersson et al. 2022; Howat et al. 2014; Jin et al. 2024; Virk et al. 2019; Zhou et al. 2023), typically indicating only whether antibodies were commonly used. Although testing every available antibody was not feasible, a systematic evaluation was conducted under multiple experimental conditions and stringent controls, including knockout cells and tissues, to provide relevant and practical guidance for TRPV1 and TRPA1 research.

ICC detection of heterologous overexpression in HEK‐293 cells was successful with most antibodies (Table 2). All TRPV1 antibodies reliably detected transfected rat TRPV1, except for the Santa Cruz V1 antibody. Notably, the four effective antibodies were raised against rat TRPV1 antigens, whereas Santa Cruz V1 was generated using a human TRPV1 antigen, suggesting that species specificity is strongly influenced by the origin of the antigen. Indeed, although the exact sequence was not disclosed, Santa Cruz V1 targets the C‐terminus of human TRPV1, a region with relatively low sequence identity between human and rat (71.5% over the last 15 amino acids). In contrast, similar reasoning cannot explain the performance of TRPA1 antibodies. Only 4 out of 7 tested antibodies successfully detected heterologous human TRPA1 expression, despite all of them being developed against human TRPA1 antigens. We were unable to detect the TRPA1 protein with the Alomone, Sigma WH, or Santa Cruz A1 antibodies. The Alomone antibody has been widely used in various immunochemistry applications (Conklin et al. 2019; Gao et al. 2025; Meng et al. 2015; Virk et al. 2019; Yamamoto et al. 2015). However, in most reports where images of presumed TRPA1^−^ cells are shown, strong nonspecific staining is evident, making it difficult to clearly distinguish positive from negative cells. Consistent with our findings, another TRPA1 antibody validation study reported similar results with the Alomone antibody (Virk et al. 2019). For Sigma WH, we found only a single published report (Vetter et al. 2012). Santa Cruz A1 has been cited a few times (Piciu et al. 2024; Walker et al. 2025; Xia et al. 2023). In one case (Piciu et al. 2024), the staining appeared convincing, but only a single cell image was provided. In the other reports, poor image quality precluded a clear assessment of specificity. Interestingly, another study (Virk et al. 2019) tested Santa Cruz A1 antibody in transfected HEK cells with excellent results. The full protocol was not provided, but we like to point out that methanol rather than PFA was used for fixation. This raises the possibility that the performance of Santa Cruz A1 may be highly sensitive to assay conditions, such as fixation.

**TABLE 2:** Antibody performance with the different techniques used.

Antibody	Target	HEK‐293 cells		Mouse DRG	
		WB	ICC	ICC	IHC
Abcam	TRPV1	++	++	+	+
Millipore V1		−	+	+	+
Santa Cruz V1		−	−	NT	NT
Neuromics GT		−	++	−	−
Neuromics GP		++	++	+	+
Aviva	TRPA1	−	+	−	−
Novus		++	++	−	−
Millipore A1		−	+	−	−
Alomone		−	−	NT	NT
Proteintech		−	++	−	−
Sigma WF		−	−	NT	NT
Santa Cruz A1		−	−	NT	NT

*Note:* − poor, + regular, ++ good.

Abbreviations: ICC, immunocytochemistry; IHC, immunohistochemistry; NT, not tested; WB, western blotting.

Positive western blot results were more limited, as strong and well‐defined bands were detected only with the Abcam and Neuromics GP antibodies for TRPV1 and with the Novus antibody for TRPA1. Notably, these two TRPV1 antibodies corresponded to those with the highest SR values, further supporting their reliability. Similarly, the Novus antibody for TRPA1 also showed a high SR and has been previously validated for WB (Virk et al. 2019). Taken together, these findings indicate that, even when using overexpressed channels from the same species used to generate the antibodies, reliable detection by WB can be challenging and may require careful optimization of the experimental conditions.

Detecting endogenous TRPV1 and TRPA1 expression in mouse DRG neurons is inherently more challenging than identifying heterologous overexpression. This difficulty arises both from the typically lower channel abundance under physiological conditions and from the fact that most commercial antibodies, including those tested here, were raised against rat (for TRPV1) or human (for TRPA1) antigens. Although the precise target regions were provided only for some antibodies, sequence identity between species varied considerably, ranging from 69.2% to 92.8% (see Table 1). Three of the four TRPV1 antibodies that performed well in ICC of transfected cells also recognized TRPV1 in ICC of cultured mouse DRG neurons and in IHC of DRG slices (Table 2), although SR values were lower and visual discrimination between TRPV1^+^ and TRPV1^−^ neurons proved more difficult. Several published reports described similar outcomes for the Abcam and Neuromics GP antibodies in different tissues, with evident unspecific fluorescence but still with distinguishable TRPV1^+^ cells (Grlickova‐Duzevik et al. 2023; Liu et al. 2016; Oh et al. 2023; Wadachi and Hargreaves 2006; Xing et al. 2019; Yoshikawa et al. 2022; Zhang et al. 2023, 2021). The Millipore antibody generated stronger background, particularly at higher concentrations, but has been reported to perform well in rat tissue (Besecker et al. 2020; Jin et al. 2024), suggesting a more stringent species specificity. Interestingly, a previous validation study (Jin et al. 2024) found that Neuromics GP and Millipore antibodies produced good results in rat DRG IHC, but not in biopsies of human skin. Together with our findings, this highlights the critical importance of considering the antigen species used during antibody generation. Unfortunately, the target sequence is often not disclosed by the antibody suppliers, a detestable practice. The antigen sequence used for Neuromics GP shares 92.8% identity between mouse and rat, but only 64.3% between rat and human. This could help explain why we detected specific staining with rTRPV1 transfected cells and mouse DRG while in (Jin et al. 2024) specific staining was observed in rat but not in human samples. Neuromics GT failed to label TRPV1 in mouse DRG cells or tissue, however it has been used before in mouse DRG as well as trigeminal ganglia (TG) albeit with high background fluorescence. Moreover, we previously successfully employed this antibody to stain mouse corneal nerve bundles (Fernández‐Trillo et al. 2020) and it appears to work well in rat DRG (Lee, Zhang, et al. 2016). Considering that the Neuromics GT antigen shares only ~81% identity between rat and mouse, these observations suggest that this antibody is better suited for rat tissue and is less efficient for detecting mTRPV1.

Our combined data for the Abcam, Millipore, and Neuromics GP antibodies suggest that, although not optimal, they can be used to detect mouse TRPV1 when appropriate controls are included, careful handling is ensured, and staining protocols are properly optimized. Based on our findings, Neuromics GT anti‐TRPV1 antibody may also be suitable for some applications.

The performance of TRPA1 antibodies in mouse DRGs was rather disappointing in our hands, as none of the antibodies that successfully detected hTRPA1 in transfected HEK cells produced a signal distinguishable from background fluorescence in either cultured mouse DRG neurons or tissue slices (Table 2). Notably, all tested antibodies were generated against human TRPA1 antigens (except Proteintech whose antigen is unknown). For the Novus antibody, the exact antigen sequence is provided and shares only 77% identity with the corresponding mouse region, which may contribute to the lack of detection. A comparison of our findings with previously published data helps to highlight the challenges and limitations associated with TRPA1 antibodies. Thus, although one report claimed validation of the Aviva antibody in mouse trigeminal ganglion (Trevisan et al. 2016), the image presented shows almost all neurons stained, which contradicts mRNA expression and functional evidence (Bautista et al. 2005; Gers‐Barlag et al. 2025; Kobayashi et al. 2005; Story et al. 2003). Other studies reported that this antibody failed to detect TRPA1 in HEK‐293 cells transfected with mTRPA1, in mouse DRG neurons (Hoebart et al. 2021), and in mouse osteosarcoma tissue (Hudhud et al. 2024), supporting our negative findings. The Novus antibody has been used to detect TRPA1 in a human pancreatic cell line (Kusiak et al. 2022) and human endometriosis tissue (Zhu et al. 2022). This antibody was also used in different mouse cell tissues (Kameda et al. 2019; Wang et al. 2025; Yang et al. 2022), but target specificity was not assessed. Other studies reported that this antibody did not properly stain TRPA1 in mouse osteosarcoma samples (Hudhud et al. 2024). The Millipore anti‐TRPA1 antibody has been tested previously in mouse brain (Lee, Lee, et al. 2016; Xia et al. 2019) and nodose ganglion (Huerta et al. 2023), yet the images provided do not allow for a confident assessment of specificity. Negative results for the Proteintech antibody in mouse DRG have also been documented (Xie et al. 2022). Positive staining was detected in cultured human odontoblast‐like cells (Liu et al. 2021), albeit without specificity control. TRPA1 labelling in cultured mouse mammary epithelial cells with Proteintech appeared ubiquituous (Suzuki et al. 2020), making non‐specific staining impossible to rule out.

Our results, together with previously published data, suggest that TRPA1 antibodies may perform reliably in human samples when appropriate staining protocols are used. However, because these antibodies were generated against human TRPA1 antigens, they are probably less suitable for detecting the mouse protein. Unfortunately, most commercially available anti‐TRPA1 antibodies are based on human immunogens. As stated before, it is worth noting that non‐commercial TRPA1 antibodies successfully detecting murine TRPA1 have been developed using human immunogens (Stokes et al. 2006) and validated with TRPA1 KO mouse (Uchiyama et al. 2020), demonstrating that it is possible to detect mouse TRPA1 with human‐based antibodies. At present, several anti‐TRPA1 antibodies generated using mouse immunogens are commercially available. However, based on the published literature, many of these antibodies appear to be unreliable. For example, KM120 (Med. Chem. Pharm), cited in two studies, shows staining that appears homogeneous and nonspecific (Nakashimo et al. 2010). Similarly, Novus NB‐91319 has only a single reported ICC citation, which shows widespread staining in dorsal root ganglion neurons that is inconsistent with functional and RNA expression data (Adam et al. 2019). In addition, AA 10150‐1120 (Antibodies Online) is listed on the vendor's webpage with a citation to an article that actually reports the antibody fails to specifically label TRPA1 (Nagy et al. 2018).

Immunodetection methods vary widely in the literature, with countless protocol modifications reported. Key parameters such as fixation, tissue preparation, processing, antibody concentration, antigen retrieval, permeabilization, blocking of non‐specific binding, signal amplification, and detection strategy can all influence the outcome (Ramos‐Vara and Miller 2014). Because immunolabeling protocols vary between laboratories, we applied a standardized procedure for all antibodies tested but did not systematically explore alternative conditions. It is therefore possible that different experimental settings could yield improved results for some of the antibodies evaluated. For these reasons, our results should not be interpreted as definitive functional validations. Nonetheless, we think they provide a useful reference for selecting the most suitable antibodies for specific applications and species, and will help increase the reproducibility of published research.

A summary of the performance of all antibodies tested across the different methodologies used in this study is presented in Table 2.

## Author Contributions

**M. de las Casas:** investigation, writing – review and editing, formal analysis, methodology, visualization, validation. **R. Torres‐Montero:** investigation, methodology. **E. De la Peña:** investigation, methodology, validation, visualization, writing – review and editing, formal analysis, project administration, funding acquisition, supervision, resources. **F. Viana:** resources, project administration, formal analysis, supervision, writing – review and editing, funding acquisition. **A. Gomis:** project administration, writing – review and editing, formal analysis, funding acquisition, supervision. **P. Hernández‐Ortego:** investigation, writing – review and editing, validation, methodology, formal analysis. **J. Fernández‐Trillo:** conceptualization, investigation, methodology, validation, visualization, writing – original draft, writing – review and editing, project administration, formal analysis, data curation, supervision.

## Funding

This work was supported by grant PID2022‐140961OB‐100 funded by MCIN/AEI/10.13039/501100011033 and ERDF, PROMETEO program (PROMETEO/2021/031 and CIPROM/2024/46) from Generalitat Valenciana Government. The Instituto de Neurociencias is a “Center of Excellence Severo Ochoa” (CEX2021‐001165‐S funded by MCIN/Agencia Estatal de Investigación (AEI)/10.13039/501100011033).

## Conflicts of Interest

The authors declare no conflicts of interest.

## Supporting information


**Figure S1:** Uncropped blots corresponding to all the WB performed in this study. All replicates are included. Blue dashed boxes indicate the regions displayed in Figure 3. Yellow triangles indicate positive bands for the TRPV1/TRPA1 antibody tested. Green triangles indicate bands corresponding to the detection of TRPV1‐EYFP or TRPA1‐tGFP with anti‐GFP or anti‐tGFP antibodies confirming the expression of the fusion proteins.
**Figure S2:** Representative confocal immunofluorescence images corresponding to the antibody dilutions not shown in Figures 1, 2, 4, and 5. The complementary dilution is shown only for experiments in which the specificity ratio (SR) differed significantly between dilutions. (A) ICC of HEK293 cells transfected with rat TRPV1‐EYFP. (B) ICC of cultured DRG cells from TRPV1 EGFP mice. (C) IHC of DRG sections from TRPV1‐EGFP mice. (D) ICC of HEK293 cells transfected with human TRPA1‐tGFP.
**Figure S3:** Immunofluorescence of endogenously expressed TRPV1 in cultured DRG cells and slices from the TRPV1‐KO mouse. (A–C) Immunocytochemistry. (D–F) Immunohistochemistry. (A–C) Confocal images of cultured DRG neurons and DRG tissue sections (D–F) from TRPV1‐KO mice. EGFP (green) TRPV1 antibody (magenta), and βIII‐Tubulin (cyan). Scale bar: 50 μm. Note the homogeneous, non‐specific staining in all cases. (A–F) For each antibody and dilution, 4 pictures from 2 different animals were studied.
**Figure S4:** Comparison of the fluorescence variance between TRPV1‐EGFP and TRPV1 KO cells. Specific antibodies produce a heterogeneous signal in TRPV1‐EGFP mice, where some cells exhibit strong labeling while others remain weak or negative, resulting in a higher variance across the field. In contrast, in TRPV1‐KO tissue, and assuming the absence of off‐target antibody binding, all cells are expected to display similar background fluorescence levels, resulting in lower variance. (A) Immunocytochemistry and (B) Immunohistochemistry TRPV1 staining fluorescence variance in TRPV1‐EGFP (filled circles) and TRPV1‐KO (open circles) microimages, using 1:500 (light blue) or 1:200 (dark blue) dilution of the indicated antibody. Each circle corresponds to the variance of the log‐normalized fluorescence quantified in all cells from a microscopic field. Bar histogram summarizes the median variance and error bars the 95% confidence interval. For each antibody and dilution, a minimum of 92 cells were analyzed across 4 microscopic fields from 2 different animals. *p < 0.05 Mann–Whitney test.
**Figure S5:** Functional characterization of TRPA1‐Cre‐ChR2‐EYFP mice. Representative traces of calcium transients evoked by AITC in the same DRG neurons than in Figure 6 but differently color coded. Traces corresponding to EYFP^+^ neurons are displayed in green and EYFP^−^ cells are displayed in blue. The pie charts represent the proportion of neurons (EYFP^+^ or EYFP^−^) responding or failing to respond to 50 μM AITC. Data obtained from 424 cells from 5 coverslips.

**Data S1:** jnc70444‐sup‐0002‐DataS1.xlsx.

## Data Availability

The datasets generated during and/or analyzed during the current study are available from the corresponding author on reasonable request.
