# Supplementary material for: Validation of TRPA1 and TRPV1 Antibodies for Expression Detection in Mammalian Cells and Tissues
Source: J Neurochem. 2026 Apr 22;170:e70444. doi: 10.1111/jnc.70444 (PMC13100940; doi:10.1111/jnc.70444)
Supplement: Supplementary file 1 — Figure S1: Uncropped blots corresponding to all the WB performed in this study. All replicates are included. Blue dashed boxes indicate the regions displayed in Figure 3. Yellow triangles indicate positive bands for the TRPV1/TRPA1 antibody tested. Green triangles indicate bands corresponding to the detection of TRPV1‐EYFP or TRPA1‐tGFP with anti‐GFP or anti‐tGFP antibodies confirming the expression of the fusion proteins. Figure S2: Representative confocal immunofluorescence images corresponding to the antibody dilutions not shown in Figures 1, 2, 4, and 5. The complementary dilution is shown only for experiments in which the specificity ratio (SR) differed significantly between dilutions. (A) ICC of HEK293 cells transfected with rat TRPV1‐EYFP. (B) ICC of cultured DRG cells from TRPV1 EGFP mice. (C) IHC of DRG sections from TRPV1‐EGFP mice. (D) ICC of HEK293 cells transfected with human TRPA1‐tGFP. Figure S3: Immunofluorescence of endogenously expressed TRPV1 in cultured DRG cells and slices from the TRPV1‐KO mouse. (A–C) Immunocytochemistry. (D–F) Immunohistochemistry. (A–C) Confocal images of cultured DRG neurons and DRG tissue sections (D–F) from TRPV1‐KO mice. EGFP (green) TRPV1 antibody (magenta), and βIII‐Tubulin (cyan). Scale bar: 50 μm. Note the homogeneous, non‐specific staining in all cases. (A–F) For each antibody and dilution, 4 pictures from 2 different animals were studied. Figure S4: Comparison of the fluorescence variance between TRPV1‐EGFP and TRPV1 KO cells. Specific antibodies produce a heterogeneous signal in TRPV1‐EGFP mice, where some cells exhibit strong labeling while others remain weak or negative, resulting in a higher variance across the field. In contrast, in TRPV1‐KO tissue, and assuming the absence of off‐target antibody binding, all cells are expected to display similar background fluorescence levels, resulting in lower variance. (A) Immunocytochemistry and (B) Immunohistochemistry TRPV1 staining fluorescence variance in TRPV1‐EGFP ( [file JNC-170-e70444-s001.pdf]

Validation of TRPA1 and TRPV1 Antibodies for Expression Detection in Mammalian Cells and Tissues

M. de las Casas, P. Hernández-Ortego, R. Torres-Montero, E. De la Peña, A. Gomis, F. Viana, J. Fernández-Trillo

Supplementary Figures

Figure S1

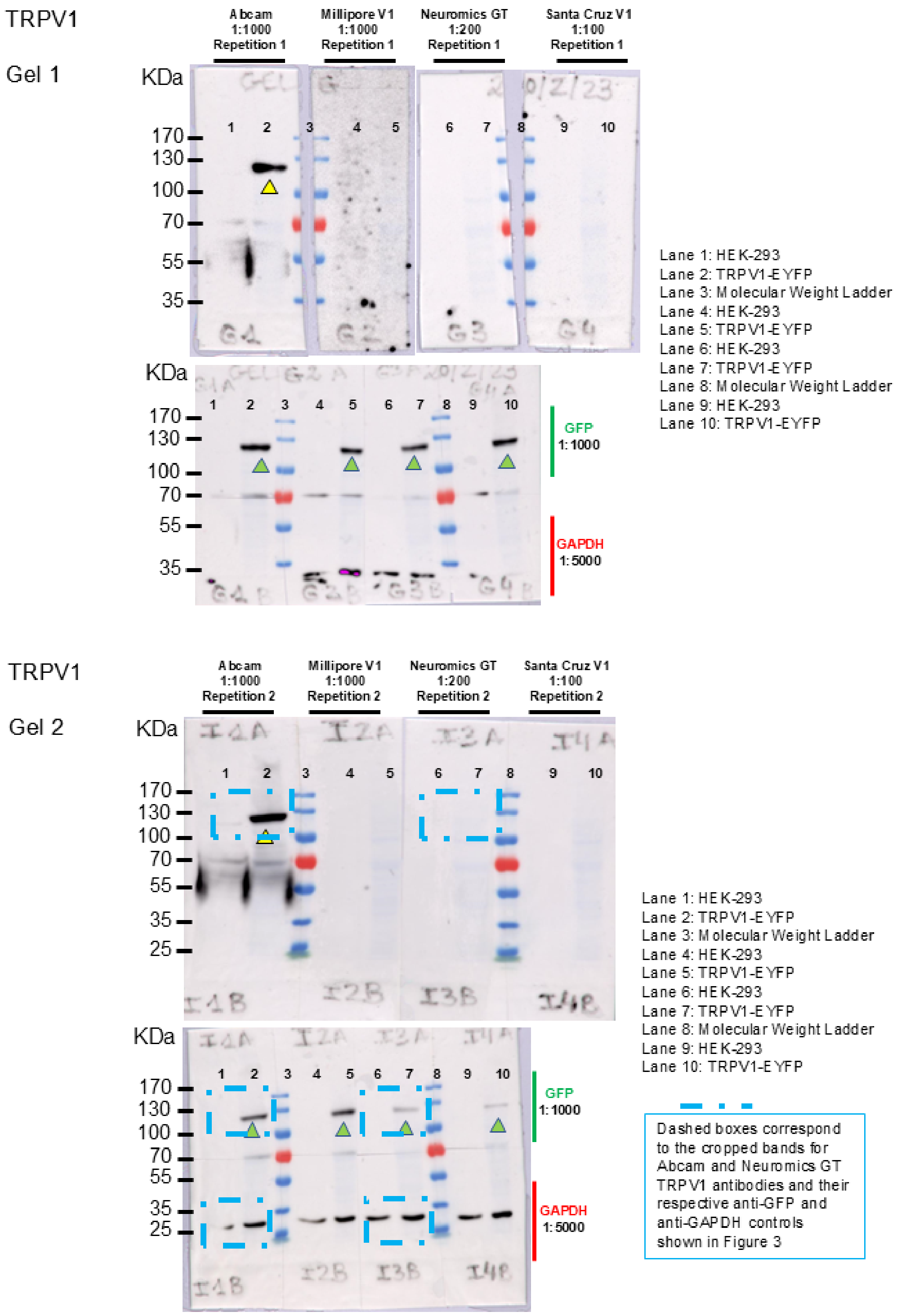

Figure S1 (cont.)

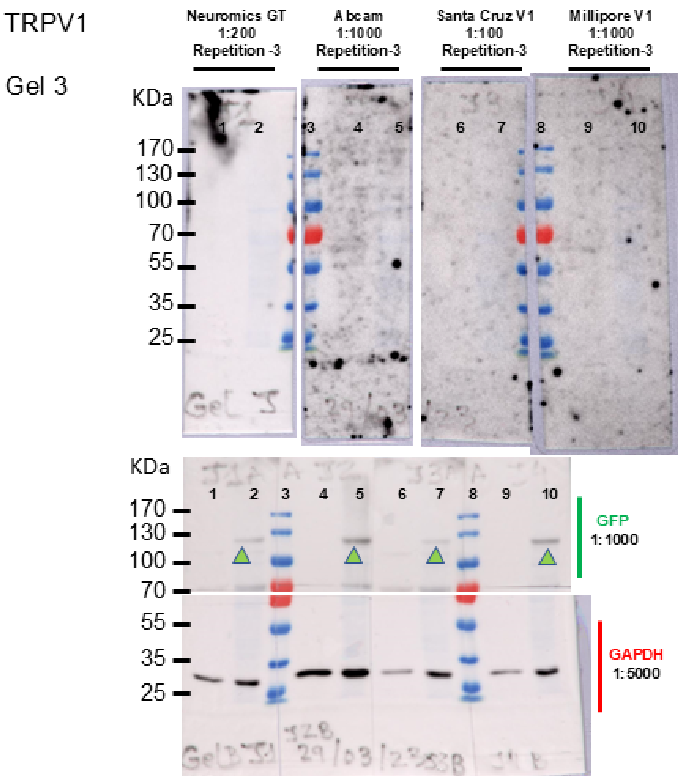

Lane 1: HEK-293
Lane 2: TRPV1-EYFP
Lane 3: Molecular Weight Ladder
Lane 4: HEK-293
Lane 5: TRPV1-EYFP
Lane 6: HEK-293
Lane 7: TRPV1-EYFP
Lane 8: Molecular Weight Ladder
Lane 9: HEK-293
Lane 10: TRPV1-EYFP

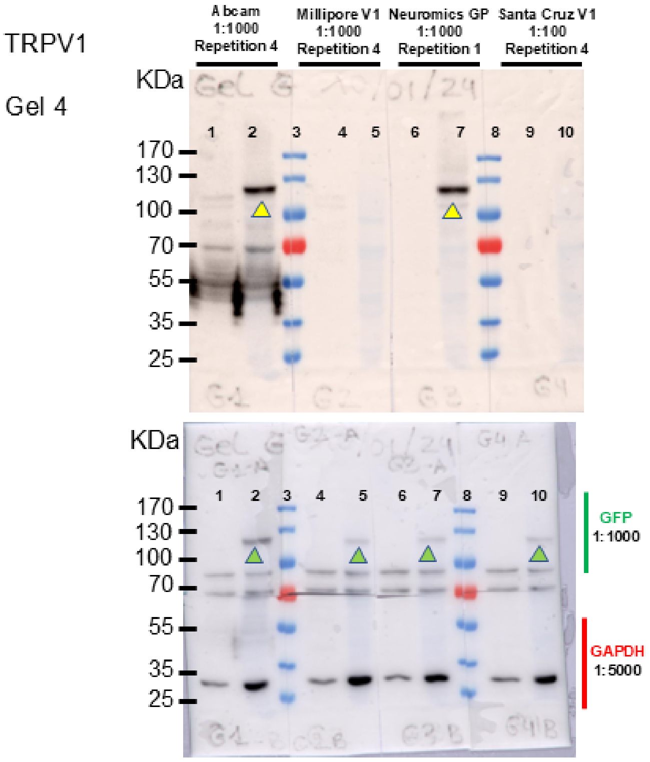

Lane 1: HEK-293
Lane 2: TRPV1-EYFP
Lane 3: Molecular Weight Ladder
Lane 4: HEK-293
Lane 5: TRPV1-EYFP
Lane 6: HEK-293
Lane 7: TRPV1-EYFP
Lane 8: Molecular Weight Ladder
Lane 9: HEK-293
Lane 10: TRPV1-EYFP

Figure S1 (cont.)

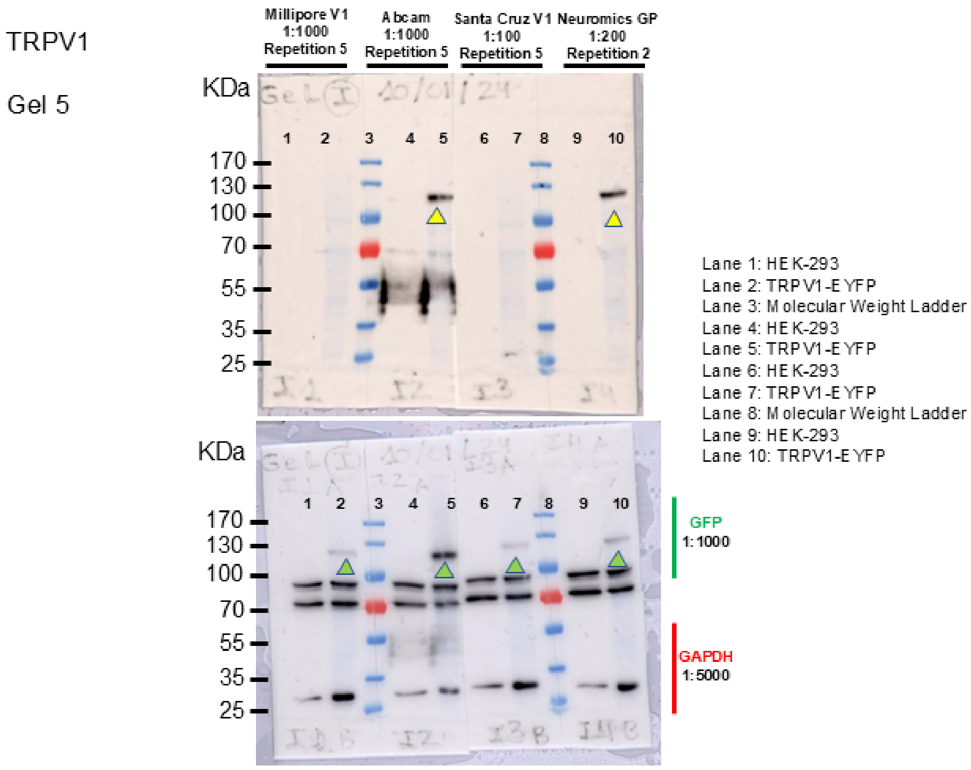

Lane 1: HEK-293
Lane 2: TRPV1-EYFP
Lane 3: Molecular Weight Ladder
Lane 4: HEK-293
Lane 5: TRPV1-EYFP
Lane 6: HEK-293
Lane 7: TRPV1-EYFP
Lane 8: Molecular Weight Ladder
Lane 9: HEK-293
Lane 10: TRPV1-EYFP

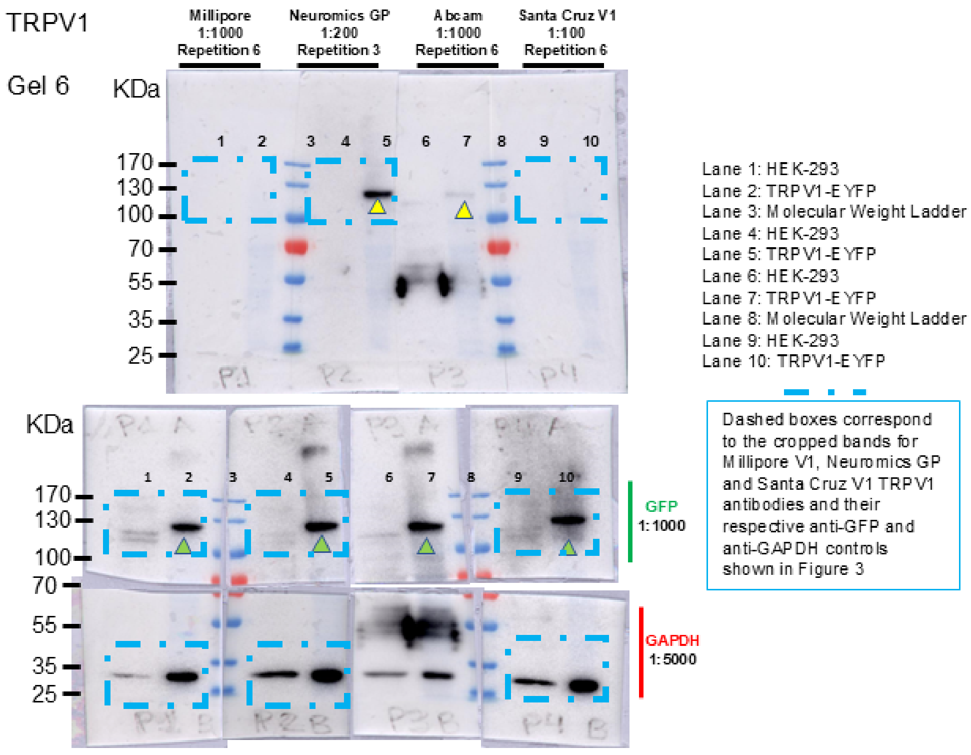

Lane 1: HEK-293
Lane 2: TRPV1-EYFP
Lane 3: Molecular Weight Ladder
Lane 4: HEK-293
Lane 5: TRPV1-EYFP
Lane 6: HEK-293
Lane 7: TRPV1-EYFP
Lane 8: Molecular Weight Ladder
Lane 9: HEK-293
Lane 10: TRPV1-EYFP

Dashed boxes correspond to the cropped bands for Millipore V1, Neuromics GP and Santa Cruz V1 TRPV1 antibodies and their respective anti-GFP and anti-GAPDH controls shown in Figure 3

Figure S1 (cont.)

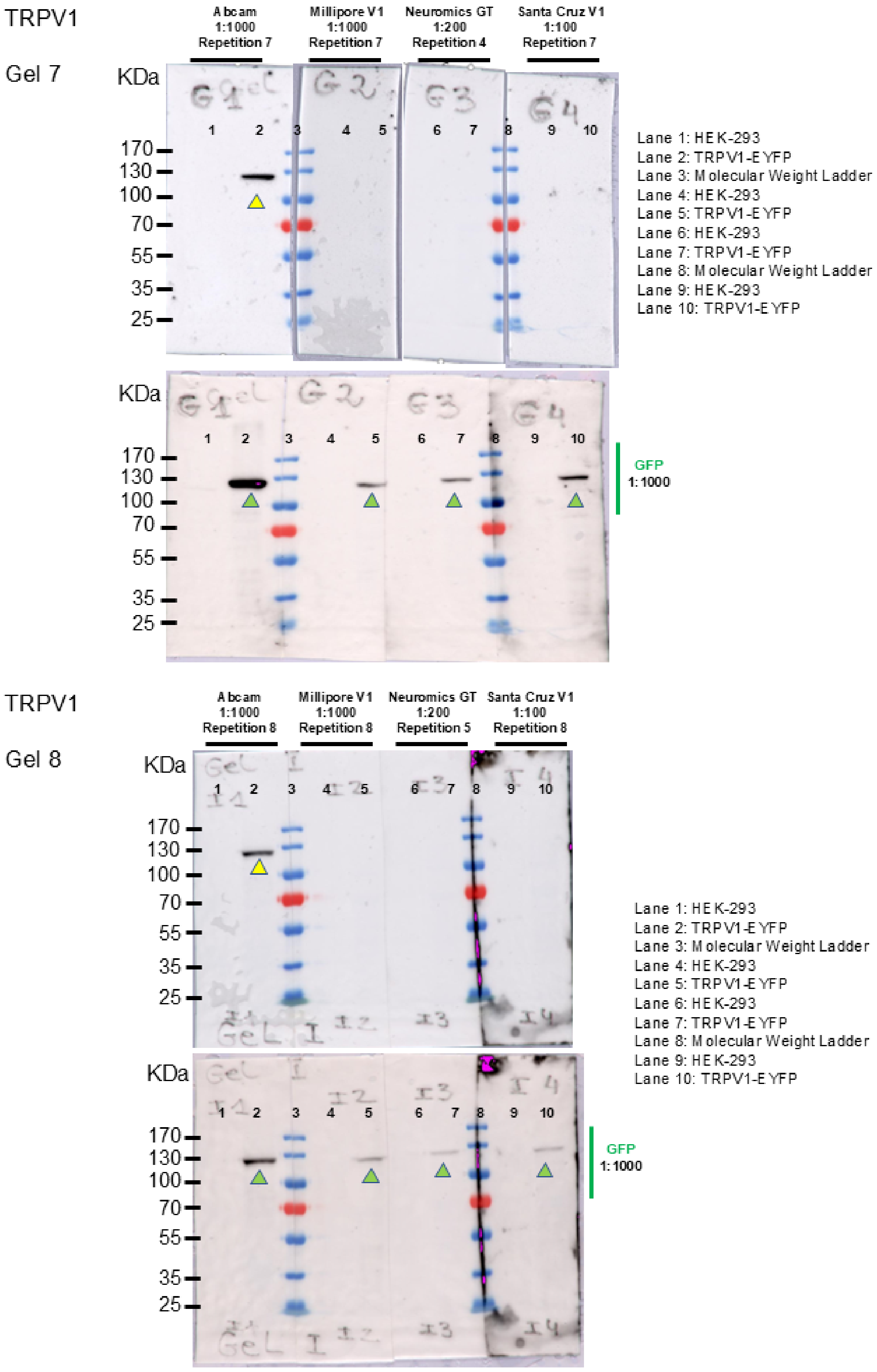

Figure S1 (cont.)

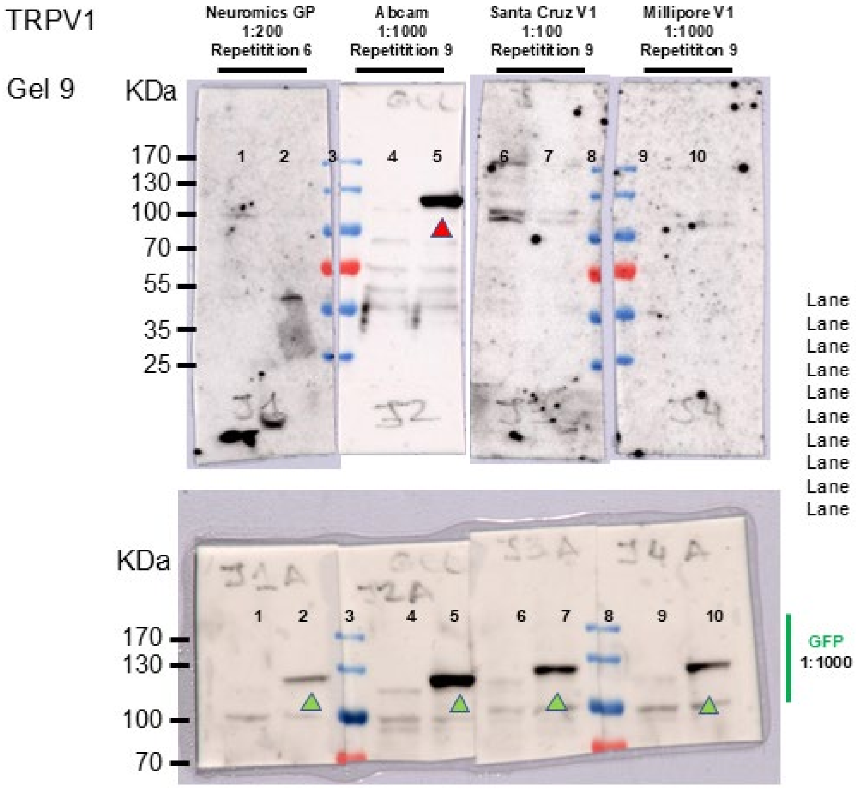

Lane 1: HEK-293
Lane 2: TRPV1-EYFP
Lane 3: Molecular Weight Ladder
Lane 4: HEK-293
Lane 5: TRPV1-EYFP
Lane 6: HEK-293
Lane 7: TRPV1-EYFP
Lane 8: Molecular Weight Ladder
Lane 9: HEK-293
Lane 10: TRPV1-EYFP

Figure S1 (cont.)

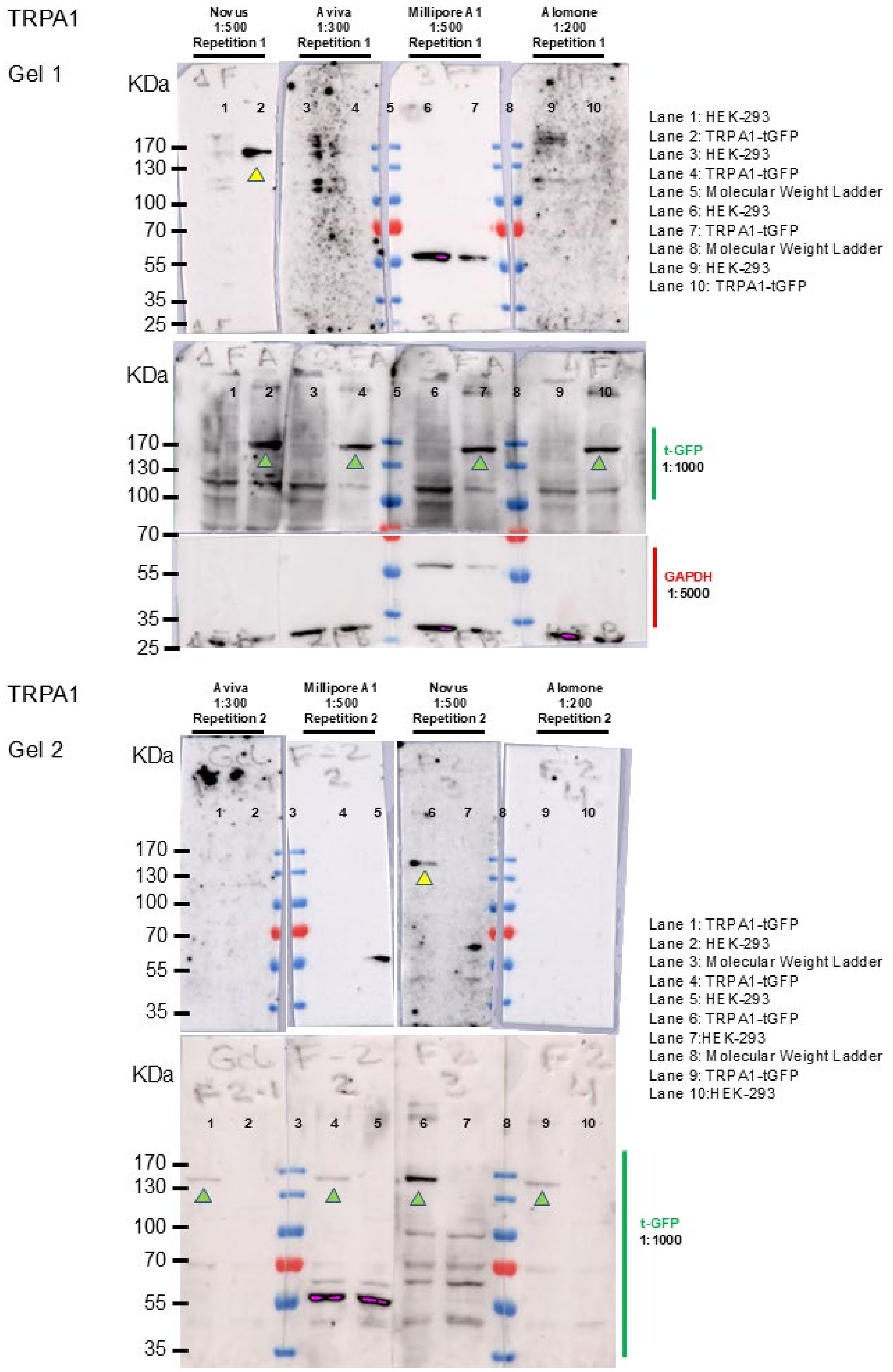

Figure S1 (cont.)

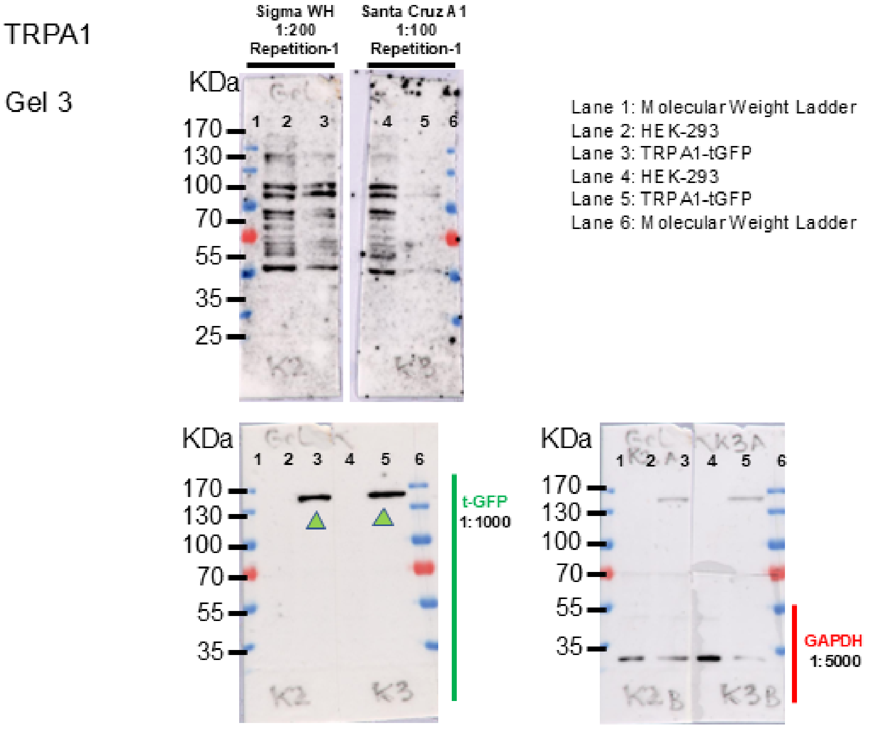

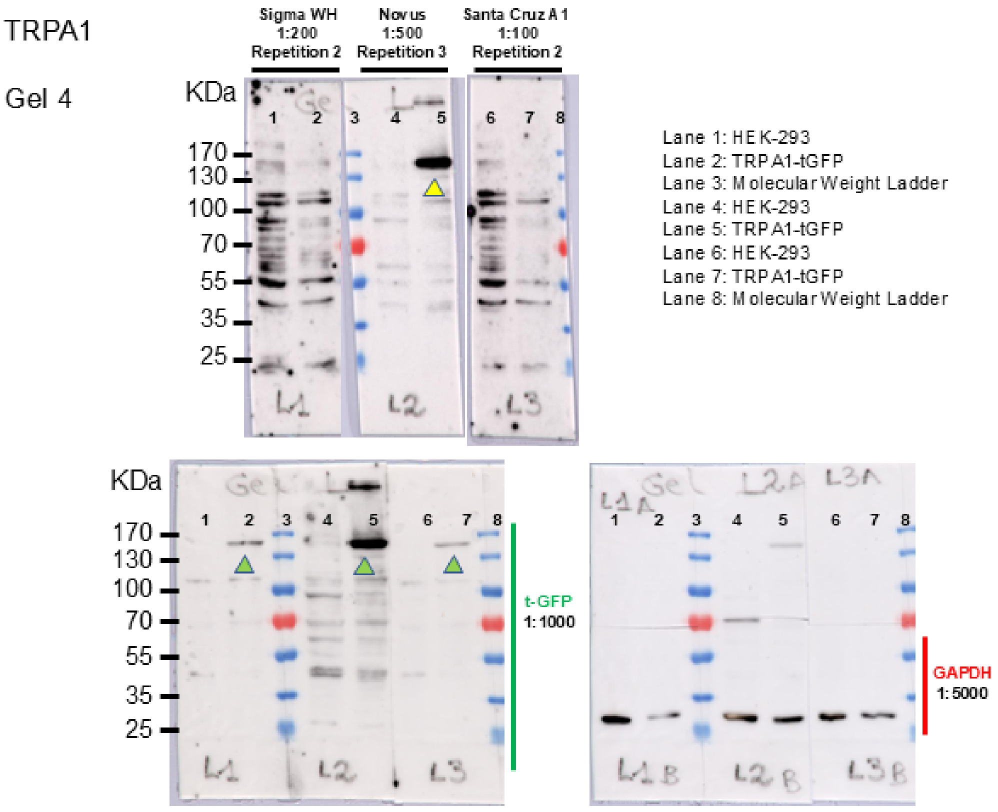

Figure S1 (cont.)

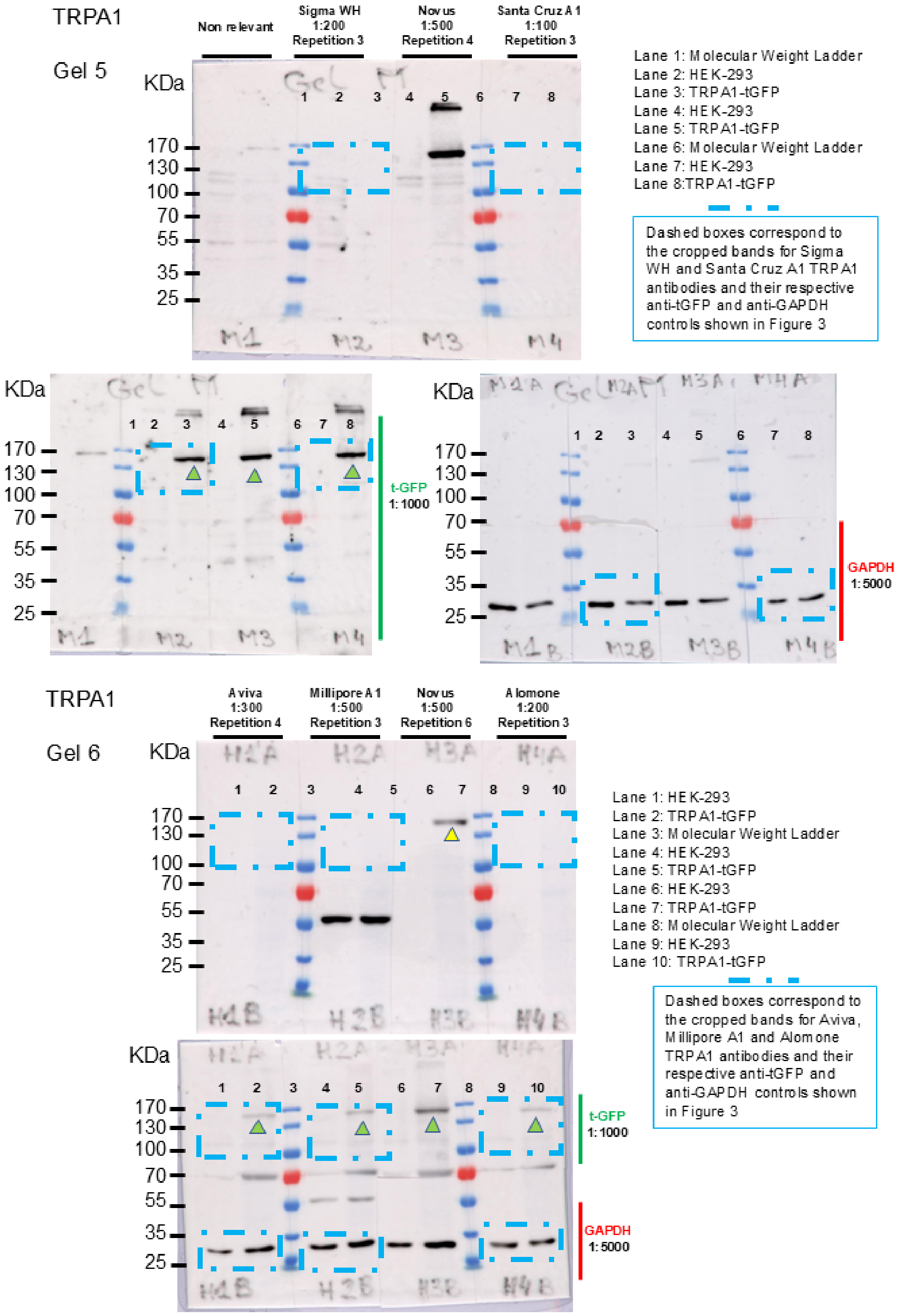

Figure S1 (cont.)

TRPA1

Gel 7

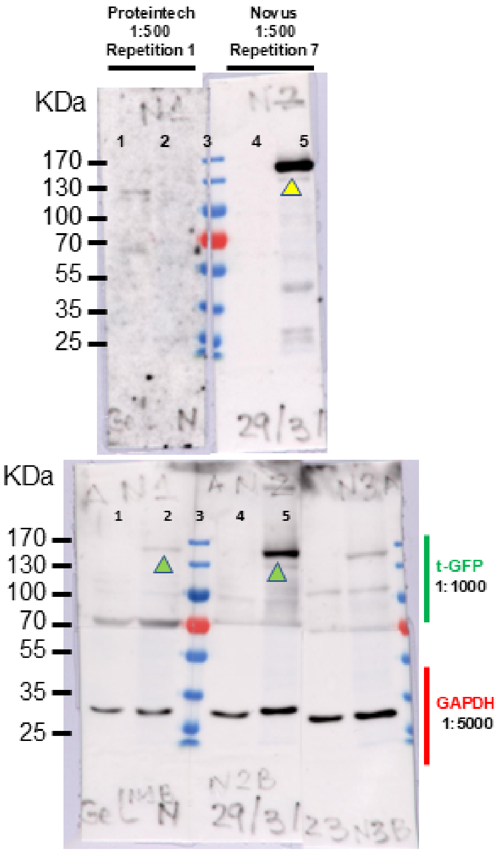

Lane 1: HEK-293
Lane 2: TRPA1-tGFP
Lane 3: Molecular Weight Ladder
Lane 4: HEK-293
Lane 5: TRPA1-tGFP

TRPA1

Gel 8

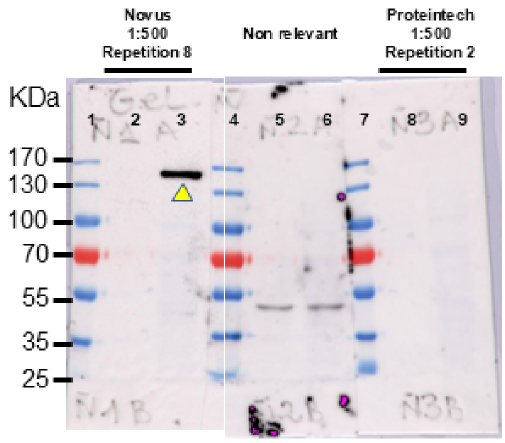

Lane 1: Molecular Weight Ladder
Lane 2: HEK-293
Lane 3: TRPA1-tGFP
Lane 4: Molecular Weight Ladder
Lane 5: HEK-293
Lane 6: TRPA1-tGFP
Lane 7: Molecular Weight Ladder
Lane 8: HEK-293
Lane 9: TRPA1-tGFP

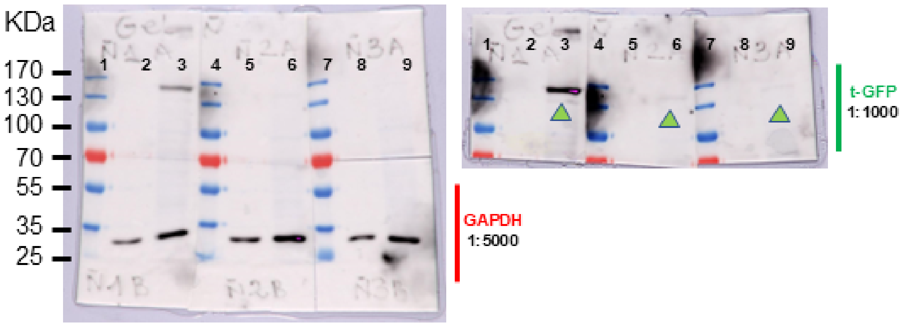

Figure S1 (cont.)

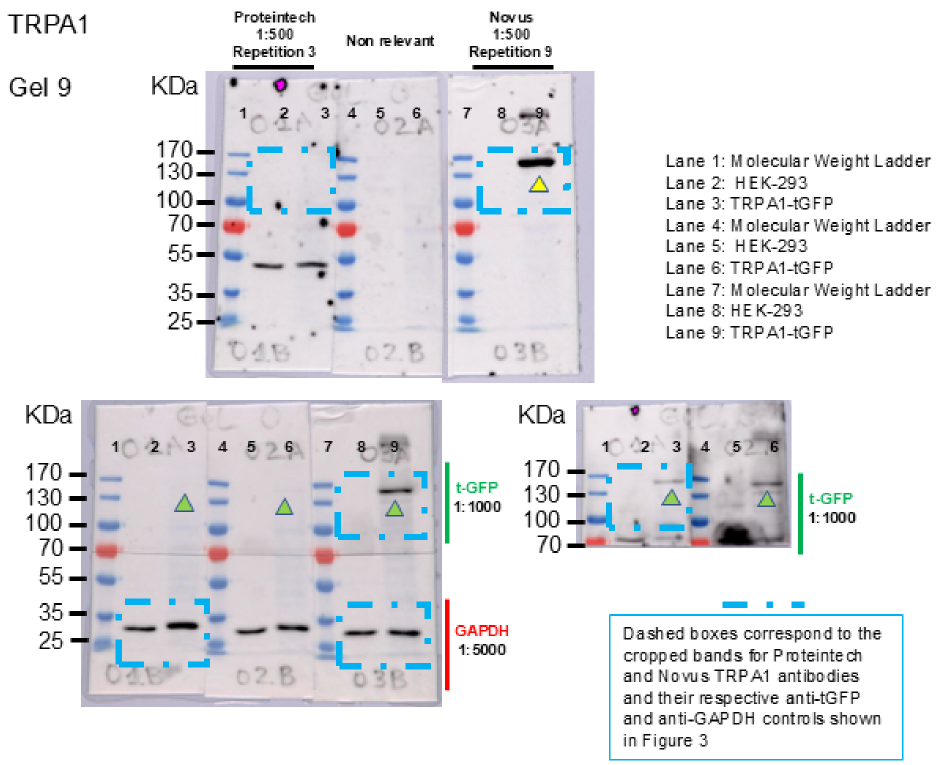

Figure S1. Uncropped blots corresponding to all the WB performed in this study. All replicates are included. Blue dashed boxes indicate the regions displayed in Figure 3. Yellow triangles indicate positive bands for the TRPV1/TRPA1 antibody tested. Green triangles indicate bands corresponding to the detection of TRPV1-EYFP or TRPA1-tGFP with anti-GFP or anti-tGFP antibodies confirming the expression of the fusion proteins.

Figure S2

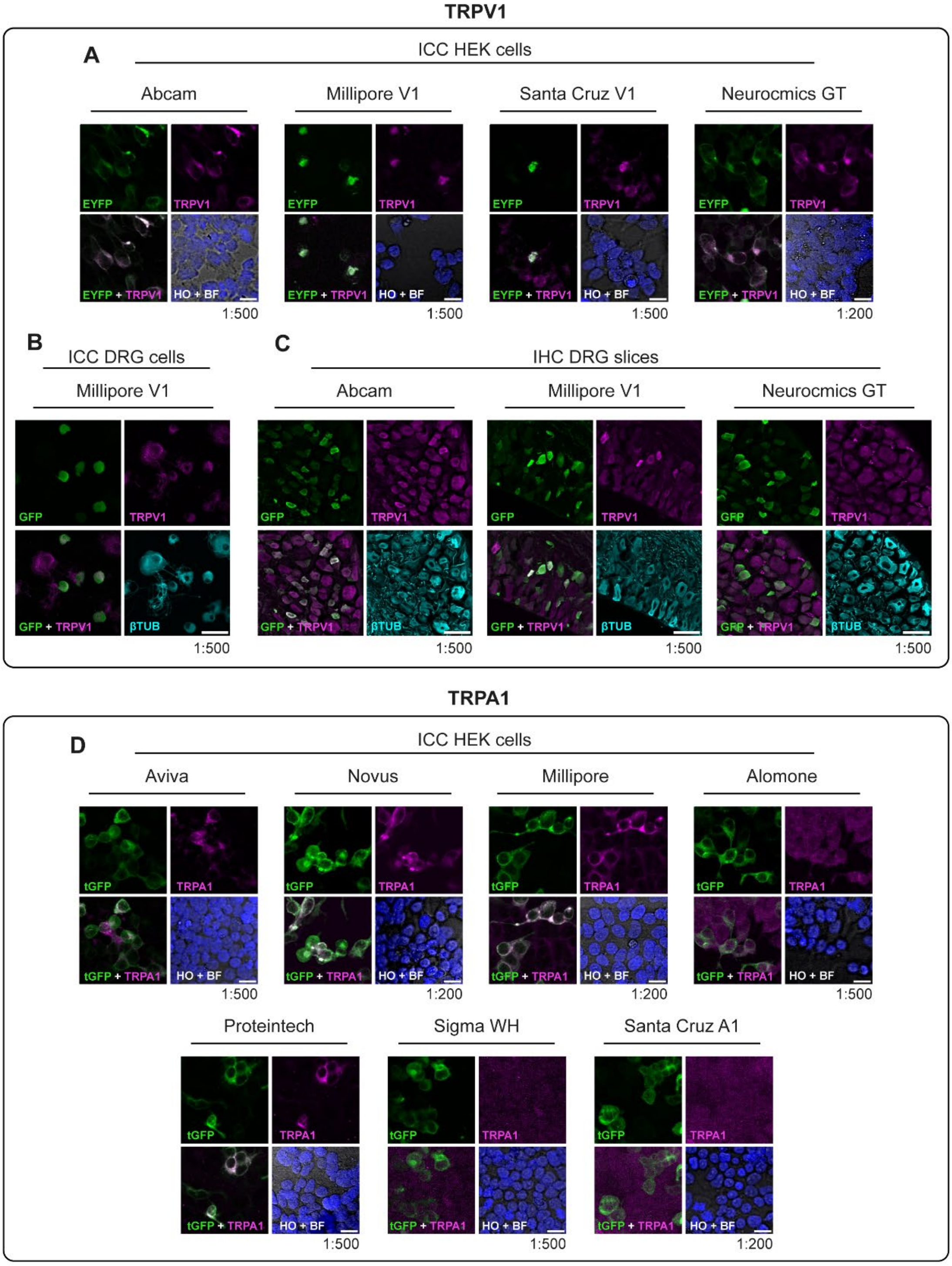

Figure S2. Representative confocal immunofluorescence images corresponding to the antibody dilutions not shown in Figures 1, 2, 4, and 5. The complementary dilution is shown only for experiments in which the specificity ratio (SR) differed significantly between dilutions. (A) ICC of HEK293 cells transfected with rat TRPV1-EYFP. (B) ICC of cultured DRG cells from TRPV1-EGFP mice. (C) IHC of DRG sections from TRPV1-EGFP mice. (D) ICC of HEK293 cells transfected with human TRPA1-tGFP.

Figure S3

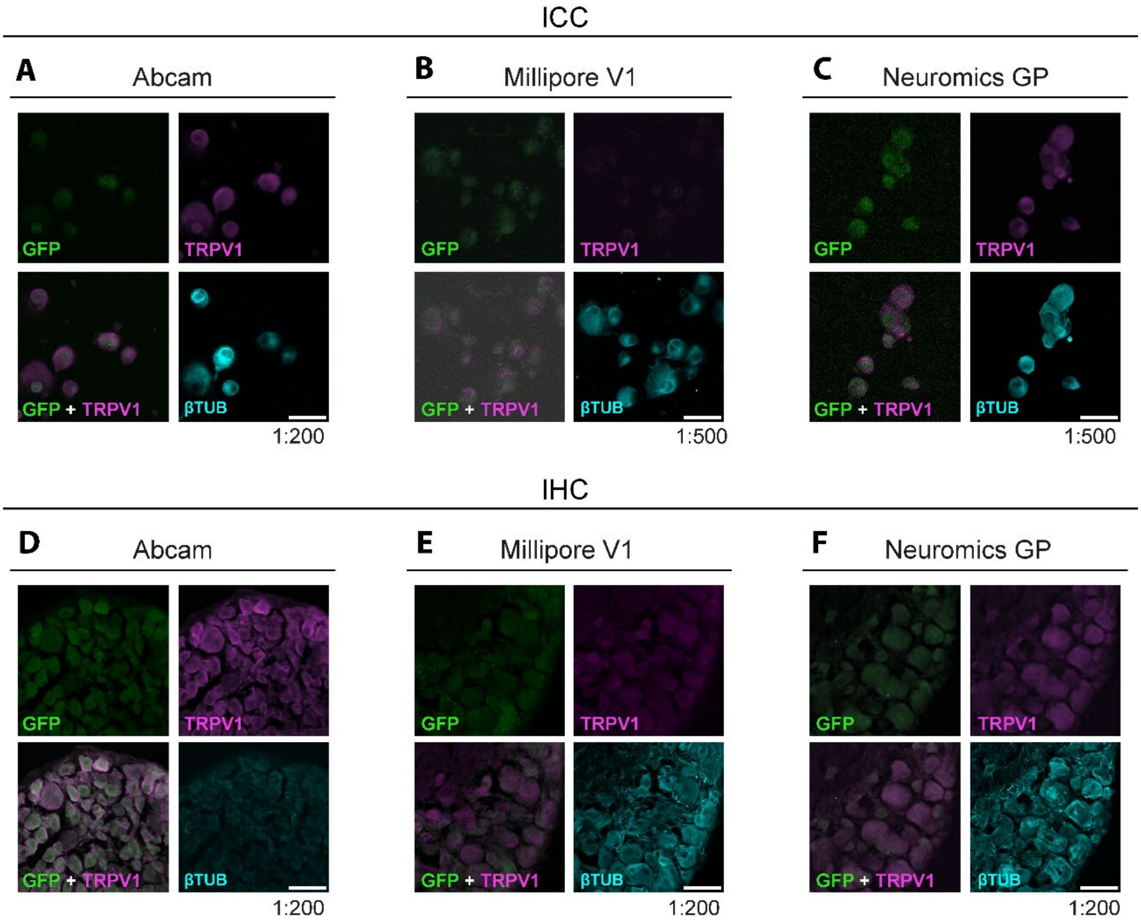

Figure S3. Immunofluorescence of endogenously expressed TRPV1 in cultured DRG cells and slices from the TRPV1-KO mouse. (A-C) Immunocytochemistry. (D-F) Immunohistochemistry. (A-C) Confocal images of cultured DRG neurons and DRG tissue sections (D-F) from TRPV1-KO mice. EGFP (green) TRPV1 antibody (magenta), and βIII-Tubulin (cyan). Scale bar: 50 µm. Note the homogeneous, non-specific staining in all cases. (A-F) For each antibody and dilution, 4 pictures from 2 different animals were studied.

Figure S4

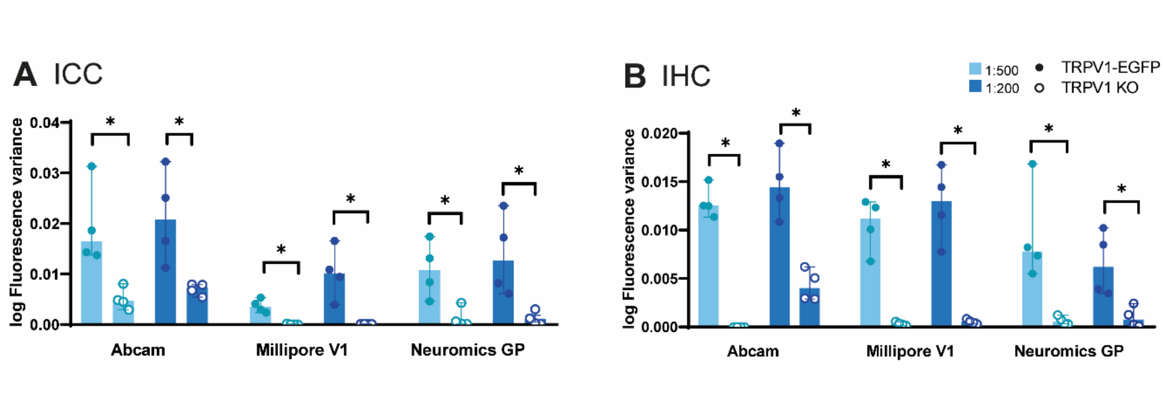

Figure S4. Comparison of the fluorescence variance between TRPV1-EGFP and TRPV1 KO cells. Specific antibodies produce a heterogeneous signal in TRPV1-EGFP mice, where some cells exhibit strong labeling while others remain weak or negative, resulting in a higher variance across the field. In contrast, in TRPV1-KO tissue, and assuming the absence of off-target antibody binding, all cells are expected to display similar background fluorescence levels, resulting in lower variance. (A) Immunocytochemistry and (B) Immunohistochemistry TRPV1 staining fluorescence variance in TRPV1-EGFP (filled circles) and TRPV1-KO (open circles) microimages, using 1:500 (light blue) or 1:200 (dark blue) dilution of the indicated antibody. Each circle corresponds to the variance of the log-normalized fluorescence quantified in all cells from a microscopic field. Bar histogram summarizes the median variance and error bars the 95% confidence interval. For each antibody and dilution, a minimum of 92 cells were analyzed across 4 microscopic fields from 2 different animals. * $p < 0.05$ Mann-Whitney test.

Figure S5

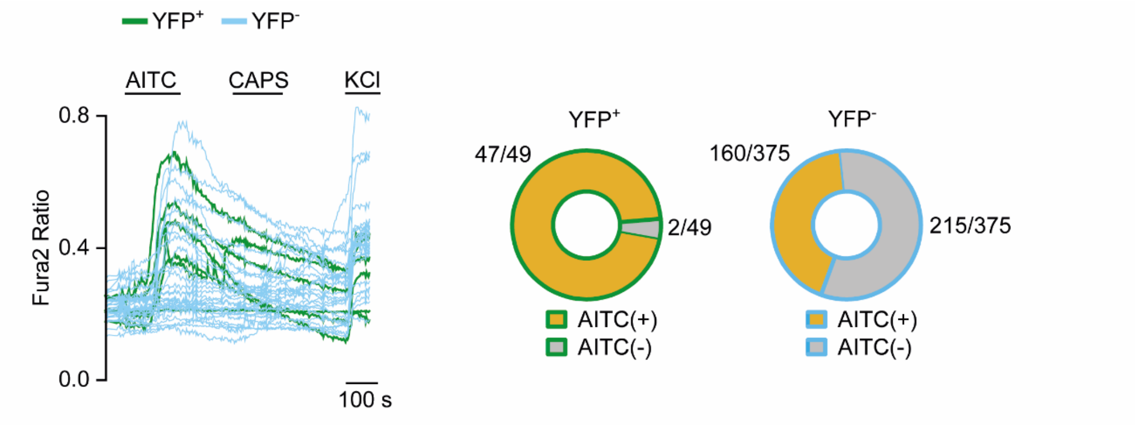

Figure S5. Functional characterization of TRPA1-Cre-ChR2-EYFP mice. Representative traces of calcium transients evoked by AITC in the same DRG neurons than in Figure 6 but differently color coded. Traces corresponding to $EYFP^+$ neurons are displayed in green and $EYFP^-$ cells are displayed in blue. The pie charts represent the proportion of neurons ($EYFP^+$ or $EYFP^-$) responding or failing to respond to 50 µM AITC. Data obtained from 424 cells from 5 coverslips.
